# Defining the Specificity of Cotranslationally Acting Chaperones by Systematic Analysis of mRNAs Associated with Ribosome-Nascent Chain Complexes

**DOI:** 10.1371/journal.pbio.1001100

**Published:** 2011-07-12

**Authors:** Marta del Alamo, Daniel J. Hogan, Sebastian Pechmann, Veronique Albanese, Patrick O. Brown, Judith Frydman

**Affiliations:** 1 Department of Biology and BioX Program, Stanford University, Stanford, California, United States of America; 2 Department of Biochemistry, Stanford University School of Medicine, Stanford, California, United States of America; 3 Howard Hughes Medical Institute, Stanford University School of Medicine, Stanford, California, United States of America; University of California San Francisco/Howard Hughes Medical Institute, United States of America

## Abstract

Polypeptides exiting the ribosome must fold and assemble in the crowded environment of the cell. Chaperones and other protein homeostasis factors interact with newly translated polypeptides to facilitate their folding and correct localization. Despite the extensive efforts, little is known about the specificity of the chaperones and other factors that bind nascent polypeptides. To address this question we present an approach that systematically identifies cotranslational chaperone substrates through the mRNAs associated with ribosome-nascent chain-chaperone complexes. We here focused on two *Saccharomyces cerevisiae* chaperones: the Signal Recognition Particle (SRP), which acts cotranslationally to target proteins to the ER, and the Nascent chain Associated Complex (NAC), whose function has been elusive. Our results provide new insights into SRP selectivity and reveal that NAC is a general cotranslational chaperone. We found surprising differential substrate specificity for the three subunits of NAC, which appear to recognize distinct features within nascent chains. Our results also revealed a partial overlap between the sets of nascent polypeptides that interact with NAC and SRP, respectively, and showed that NAC modulates SRP specificity and fidelity in vivo. These findings give us new insight into the dynamic interplay of chaperones acting on nascent chains. The strategy we used should be generally applicable to mapping the specificity, interplay, and dynamics of the cotranslational protein homeostasis network.

## Author Summary

In every cell, ribosomes translate the genetic instructions carried by messenger RNAs into the proteins they encode. Molecular midwives called chaperones often bind to nascent protein chains as they emerge from the ribosome to help them fold. Very little is known about this process. Do all proteins need chaperone assistance as they exit the ribosome? Do different chaperones recognize different polypeptide chains and, if so, how? Answering these questions has been hard because most studies have examined only a handful of model proteins and their interactions with a specific chaperone. Here, we used a systematic approach to investigate the challenging question of chaperone specificity in living cells. We isolated specific chaperones that interact with nascent proteins during translation along with the ribosomes and associated mRNAs encoding the emerging proteins. We then used DNA microarrays to identify the full suite of mRNAs and thus the encoded proteins that interact cotranslationally with each of these factors. We learned from these studies that individual chaperones interact with a specific set of nascent proteins. Furthermore, overlapping specificity enables one chaperone to modulate the specificity and fidelity of another. The picture that emerges suggests that these molecular midwives are an important part of the intricate systems that maintain specificity, precision, and efficiency in expressing the genome's instructions.

## Introduction

Ribosomes translate the linear genetic code into polypeptide chains that must fold into a specific three-dimensional structure and often assemble with other polypeptides to be born as functional proteins. During this process, as nascent proteins emerge from the ribosome, they lack information to complete their folding and are susceptible to misfolding and aggregation. A diverse set of molecular chaperones act as midwives to stabilize and facilitate the folding of newly translated polypeptides into functional proteins. Among these, Chaperones Linked to Protein Synthesis (CLIPS) [1] interact physically with ribosomes and associate cotranslationally with nascent polypeptides. In addition to folding within the cytosol, many polypeptides must be directed to various membrane-bound organelles, such as the ER and mitochondria. A number of specific targeting factors recognize nascent polypeptides before they have a chance to fold in the cytosol and deliver them to specific cellular membranes. One of the best understood mechanisms involves the cotranslational recognition of characteristic hydrophobic nascent chain segments by the Signal Recognition Particle (SRP), which facilitates proper delivery of the entire ribosome-nascent chain complex (RNC) to the ER membrane for cotranslational translocation.

The multiplicity of fates and possible interactions available to a polypeptide as it emerges from the ribosome in the eukaryotic cytosol raises a number of intriguing questions. Do all nascent chains interact with chaperones? Is there any specificity in the recognition of nascent chains by chaperones? How do cytosolic chaperones and targeting factors such as SRP discriminate among their respective substrates, and how is the fidelity of this process achieved? These questions are fundamental to understanding the mechanisms governing polypeptide fate as it emerges from the ribosome.

Much of our understanding of nascent chain interactions with chaperones or other targeting factors comes from the study of model proteins, chosen for a convenient enzymatic or structural assay for folding or translocation. As a result, the overall logic and organization of the system that mediates the critical events in delivery and birth of a nascent polypeptide as a functional protein is still a mystery. To begin to fill this gap, we developed a systematic approach to define the principles underlying the specificity of cotranslational chaperones. In the present work, we apply it to study the specificity and interplay of two important nascent-chain interacting factors: SRP and the Nascent Chain Associated Complex (NAC).

Eukaryotic SRP was initially identified as a factor for targeting proteins to the ER. SRP is a ribonucleoprotein complex comprising six proteins (in yeast Srp72, Srp68, Srp54, Sec65, Srp21, Srp14) and a non-coding RNA (scR1) [2]. SRP binds weakly to all ribosomes, even those that do not translate ER-destined proteins, by virtue of its contacts with multiple ribosomal sites. One of the contact sites, the ribosomal protein Rpl25, is also a proposed binding site for NAC [3],[4]. SRP recognizes characteristic hydrophobic sequences such as the N-terminal signal sequence (SS) and transmembrane domains (TM) in nascent polypeptides as they emerge from the ribosome. The dual recognition of ribosome and nascent chain by SRP ensures high affinity for cognate RNCs. SRP-bound RNCs are targeted to the membrane through interaction with the membrane bound SRP receptor (SR), where nascent chains bearing SS or TM domains are translocated across the ER membrane by a protein complex called the Sec61 translocon. Interestingly, the Sec61 translocon itself can also interact directly with ribosomes [5], preferentially recognizing RNCs bearing hydrophobic SS or TM regions; this might provide an SRP independent route to the ER or a proofreading mechanism for ER import. SRP-independent co- and post-translational ER targeting pathways also exist, including post-translational chaperone-assisted translocation [6] and direct ER targeting of mRNA through RNA-binding proteins (reviewed in [7]). The respective contributions of the various targeting pathways to ER import in vivo and the determinants that channel an ER-bound protein through either SRP-dependent or SRP-independent pathways are not entirely understood.

Very little is known about the function of the abundant and ubiquitous NAC complex. NAC is phylogenetically conserved across eukaryotes and archaea but is absent from prokaryotes [8]. Structural characterization of archaeal NAC indicates that its subunits must assemble in tightly folded dimers [9]. Most NAC complexes are heterodimers of two subunits, α and β, but homodimers have also been reported [10]. Yeast contains a single alpha subunit gene, *EGD2*, and two β subunit genes, *EGD1* and *BTT1*. NAC contacts Rpl25 [3] and Rpl31 [11] in close proximity to the ribosomal exit site and can crosslink to very short nascent chains [12], suggesting an early role in the birth of nascent proteins. NAC deletion causes embryonic lethality in mice, flies, and nematodes [13]–[15] but only minor growth defects in yeast [16]. Despite its abundance and conservation, the specificity and function of NAC are obscure and controversial. NAC does not associate with proteins after release from the ribosome and has no apparent chaperone activity. From in vitro experiments, NAC was initially proposed to be essential for faithful SRP-targeting of proteins to the ER [12] and preventing inappropriate association of RNCs lacking SS or TM with the translocon Sec61 [17]. This hypothesis was not supported, however, by subsequent in vitro and in vivo studies, which did not reveal aberrant translocation phenotypes in NAC-deleted strains [16],[18]. A regulatory role for NAC in mitochondrial protein import, suggested by in vitro experiments [19],[20], was not corroborated by in vivo studies [16]. Given the robustness of protein homeostasis pathways, loss of NAC could be compensated by other systems. Indeed, NAC deletions exacerbate the effect of deleting the yeast Hsp70 homolog *SSB*, leading to higher levels of ribosomal protein aggregation [21].

A number of experimental challenges have hindered progress towards understanding the robust network of chaperones and cofactors acting cotranslationally on nascent chains. Because nascent chains comprise a small, transient, and heterogeneous cellular pool of chaperone substrates, proteomic analyses are currently impractical. The high degree of redundancy within the cellular chaperone network often masks obvious loss-of-function phenotypes. Our understanding of the specificity and mechanism of cotranslationally acting chaperones comes from in vitro translation experiments using individual model proteins, and thus the generality of such experiments is hard to ascertain. To circumvent these difficulties, we developed a sensitive, systematic method for defining the substrate specificity and interplay of cotranslationally acting chaperones and other nascent chain binding and modifying factors (e.g., acetylation enzymes) in vivo. Here we used this approach to characterize the specificity of the interactions of SRP and NAC with nascent polypeptides and how the interplay between these two factors serves to modulate that specificity.

## Results

### Experimental Strategy

Cotranslationally acting chaperones recognize substrates as they emerge from ribosomes; the identity of the polypeptide substrate is determined by the mRNA programming its translation. We reasoned that we could leverage the specificity, sensitivity, and comprehensiveness of RNA identification to systematically identify the substrates of factors that associate cotranslationally with nascent polypeptides.

**Figure 1:**
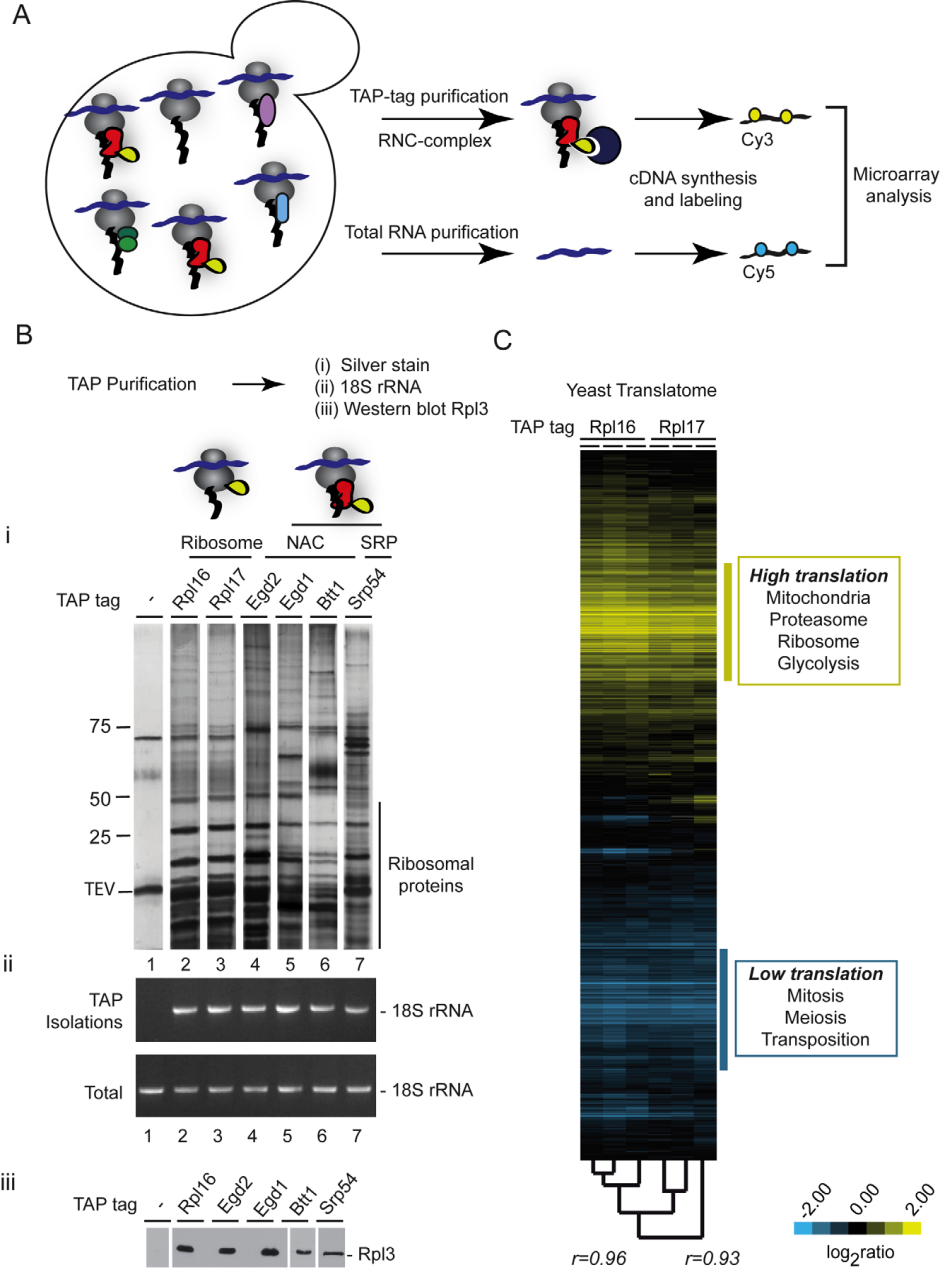
Global strategy to define specificity of ribosome-associated factors. (A) Experimental approach. Nascent polypeptides emerging from the ribosome during biogenesis can interact with many chaperones and protein homeostasis factors. The high sensitivity of RNA identification can be used to identify substrates of specific cotranslationally acting chaperones. Protein A-tagged (TAP-tag) chaperones associated with translating ribosomes were immunopurified by binding to magnetic IgG beads, washed, and chaperone complexes were eluted with TEV protease treatment. Immunopurified RNAs and total cell extract RNAs were isolated, reverse transcribed, coupled to Cy5 and Cy3 dyes, respectively, and comparatively hybridized to DNA microarrays. (B) Validation of affinity purification approach of ribosomes and ribosome-associated factors. (i) Protein profiles of affinity purified complexes. Affinity purified complexes from tagged ribosomal proteins and ribosome-associated factors were separated by SDS-PAGE and visualized by silver staining. Lane 1 corresponds to a control purification from untagged yeast cells. Main band on the negative control corresponds to the TEV protease used to elute immunopurified proteins. (ii) RT-PCR for rRNA corresponding to the 18S ribosomal subunit. Equal amounts of total RNA isolated from yeast extracts and immunopurifications proteins were reverse-transcribed with random oligonucleotides and obtained cDNA was amplified by PCR using gene-specific primers for 18S rRNA. Strains used in each lane were: 1, untagged-WT; 2, Rpl16-TAP; 3, Rpl17-TAP; 4, Egd2-TAP; 5, Egd1-TAP; 6, Btt1-TAP; 7, Srp54-TAP. (iii) Immunoblot analysis of a component of 60S ribosomal subunit. Immunopurified complexes were transferred to nitrocellulose and the Rpl3 protein was detected with a monoclonal antibody. (C) Translational profile of yeast strains derived by affinity purification of ribosomes. Hierarchically clustered heat map of the translation profiles obtained from three different immunopurifications made of both TAP-tagged Rpl16 and Rpl17 ribosomal proteins. Each column represents an experiment and each row represents a gene. Pearson correlation coefficients between experiments are indicated on the tree. Significantly enriched GO terms (*p*<0.01) are indicated.

Our basic experimental strategy was to isolate specific chaperone-bound ribosome-nascent chain complexes (RNCs) from cells and identify the polypeptide substrates through their encoding mRNAs (Figure 1A). The isolation exploits Tandem-Affinity-Purification (TAP) tagged chaperones expressed from their endogenous chromosomal locations to ensure their expression at physiological levels. Following isolation of a specific tagged chaperone and associated RNCs complexes, we can identify the mRNAs encoding the polypeptide substrates selectively bound by that chaperone, by DNA microarray hybridization (Figure 1A).

In this study, we applied this approach to define the substrate specificity of two ribosome-associated factors from the yeast *S. cerevisiae*: SRP and NAC. The multicomponent SRP complex was isolated using SRP54-TAP. To purify NAC we used TAP-tagged variants of each of the NAC subunits: Egd1/β, Egd2/α, and Btt1/β'. A similar strategy relying on C-terminal TAP-tags of two different solvent exposed ribosomal proteins, Rpl16 and Rpl17, was used to purify ribosomes directly. Sucrose gradient fractionation confirmed that the TAP-tagged Srp54, all three NAC subunits, and Rpl16 and Rpl17 all associated with polysomes (Figure S1A and Figure S6B). We initially examined the TAP-purified complexes by SDS-PAGE followed by silver staining (Figure 1Bi). Whereas the untagged purification control (Figure 1Bi, lane 1) revealed only background bands corresponding to the TEV protease preparation, all other lanes showed characteristic associated proteins (Figure 1Bi, lanes 2–7). Shared among all purifications were a set of low molecular weight proteins identified as ribosomal proteins by mass spectrometry (Figure 1Bi; unpublished data). The presence of the 40S ribosomal subunits in the TAP immunopurifications (IPs) was confirmed by RT-PCR detection of the 18S rRNA (Figure 1Bii) while immunoblot analysis for ribosomal protein Rpl3 confirmed the presence of the 60S subunit (Figure 1Biii). Importantly, neither 18S rRNA nor Rpl3 were detected in purifications carried out from untagged control cells (Figure 1Bi, lane 1). These results show that the TAP-tag does not disrupt ribosomal binding of either SRP or NAC and that our isolation procedure efficiently recovers their ribosome-associated complexes. We subsequently employed the TAP-tag isolation approach to systematically identify all mRNAs associated with SRP and the three subunits of NAC, as well as those engaged with translating ribosomes in actively growing cells.

The TAP-tags in ribosomal proteins Rpl16 or Rpl17 were used to purify all translating ribosomes irrespective of their association with chaperones (Figure 1Bi, lanes 2,3) and the associated mRNA was analyzed using DNA microarrays with the total mRNA from the same cells serving as a comparative standard (Figure 1C). The experiments were carried out as three independent biological replicates for each ribosomal protein. As shown in the clustering analysis in Figure 1C, the results of these experiments were highly reproducible (Rpl16, *r* = 0.96; between Rpl16 and Rpl17, *r* = 0.93). In principle, the relative occupancy of each mRNA with Rpl16 and Rpl17 provides a measure of that mRNA's association with translating ribosomes. At a stringent 1% false discovery rate (FDR) [22], we identified that 1,673 mRNAs are highly enriched in both Rpl16 and Rpl17 datasets. As expected, a disproportionate number of these mRNAs encode ribosomal proteins (GO “ribosome”, *n* = 212 genes, *p*<1×10^−56^), metabolic enzymes (GO “carboxylic acid metabolic process”, *n* = 199 genes, *p* = 1×10^−10^), and mitochondria (GO “mitochondrion”, *n* = 450, *p* = 1×10^−4^), which correspond to the mRNAs with the highest translation rates in actively growing cells. In contrast, the least enriched mRNAs encoded proteins likely not translated at appreciable rates in mid-log phase, including meiosis and transposition. Similar conclusions were obtained when translation was assessed in the same yeast cells by isolation of actively translated mRNAs from the polysome fractions of sucrose gradients (Figure S1); our results are also consistent with previous findings [23],[24].

### Global Identification of Cellular SRP Substrates

**Figure 2:**
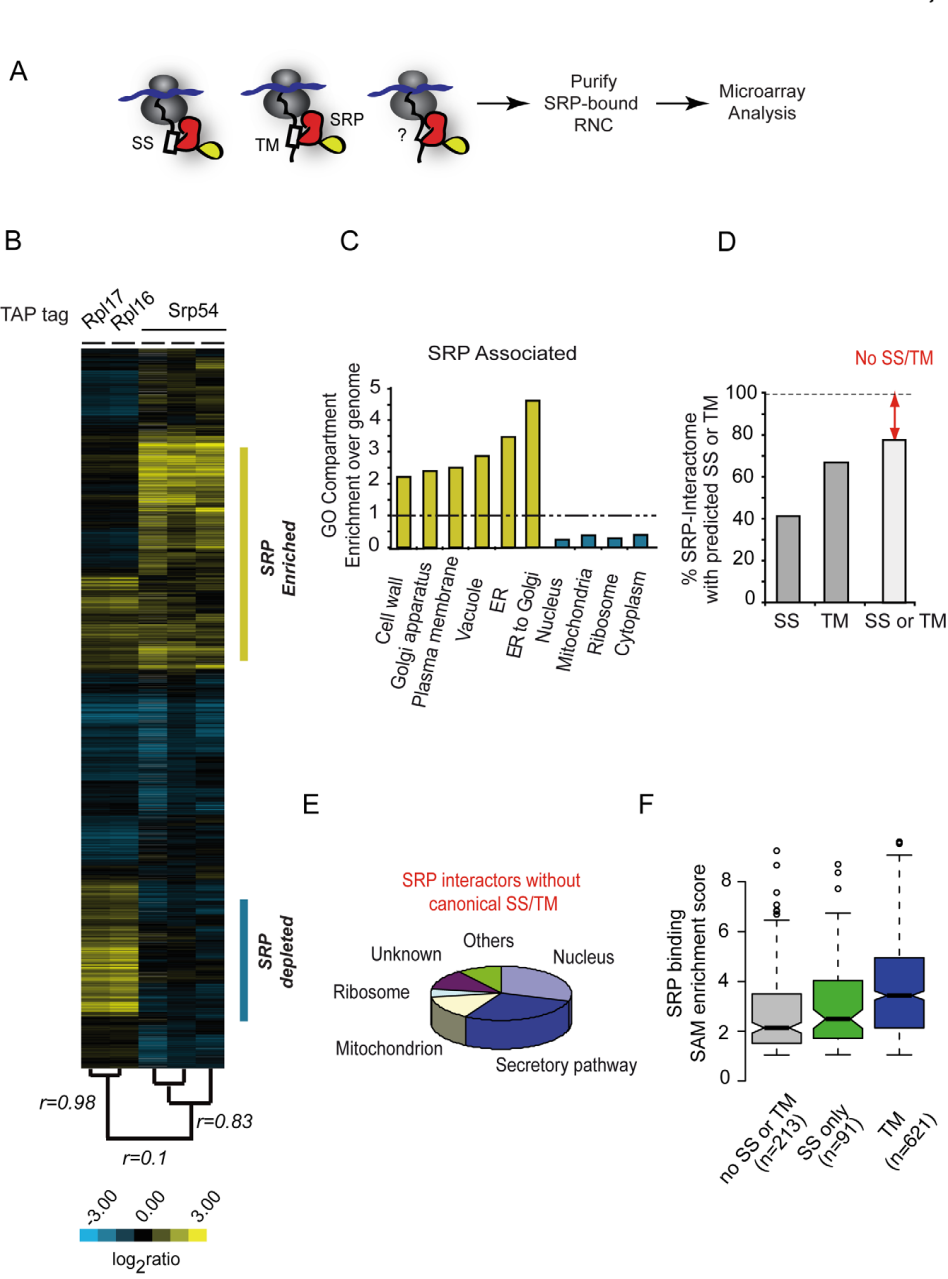
Identification and properties of polypeptides cotranslationally associated with SRP in yeast. (A) Strategy to identify SRP interactors. SRP has been described to recognize signal sequences (SS) and transmembrane regions (TM) on nascent preproteins as they emerge from the ribosome. To identify the SRP cotranslational interactome, SRP-bound ribosome-nascent chain complexes (RNC) were affinity purified and identified associated-mRNA by means of microarrays. Importantly, our procedure releases SRP from the membrane, as no ER membrane markers copurify with the SRP-RNC complexes (unpublished). (B) Translational profile of SRP-bound RNC. Hierarchically clustered heat map of the translation profiles obtained from three different immunopurifications using TAP-tagged Srp54, Rpl16, and Rpl17. Each column represents a single experiment for Srp54-TAP and the average of three independent experiments for Rpl16 and Rpl17. Pearson correlation coefficients between experiments are indicated on the tree. Genes enriched or depleted in Srp54 immunopurifications are indicated. (C) Over-represented (yellow) and under-represented (cyan) GO annotations (component) for polypeptides cotranslationally associated with SRP. The extent of the enrichment for each GO term is indicated as fold enrichment over genome. GO terms above or below the dashed line correspond to ontology categories over-represented or under-represented among SRP interacting proteins. (D) Enrichment of mRNAs encoding proteins with predicted signal sequences (SS), transmembrane regions (TM), or both in association with SRP. (E) Distribution of subcellular localization of SRP targets without canonical SS/TM. (F) Relationship between SRP binding and N-terminal features in the nascent chains. The box plot represents the distribution of the enrichment (as SAM score) obtained on the microarrays analysis for mRNAs encoding proteins with predicted signal sequences, transmembrane regions, or lacking any of these two features. The line indicates the median and the whiskers of 25%–75% of the total dataset. For details, see Material and Methods, “Enrichment Distribution Analysis.”

To identify the cellular substrates of SRP in vivo, we used immunoaffinity isolation of Srp54-TAP along with its cotranslational associated RNC-mRNAs complexes to isolate mRNAs encoding nascent proteins specifically recognized by SRP (Figure 1B, lane 7; Figure 2A). Using DNA microarrays we identified approximately 924 mRNAs reproducibly enriched at a stringent statistical threshold in Srp54 IPs (Figure 2A, note high reproducibility of three independent Srp54 replicates). Disrupting the translating 80S ribosomes with EDTA, which releases the translated mRNAs, prevented the recovery of mRNAs but not the SRP RNA scR1 in the SRP isolations (unpublished data). This indicates that the association of mRNAs with SRP was mediated through translating ribosomes, supporting our premise that analysis of the mRNAs associated with RNC-SRP complexes provides information on the specificity of SRP interaction with the translating polypeptides.

Hierarchical clustering based on quantitative enrichment of mRNAs in association with Rpl16/17 and Srp54 respectively indicated clear selectivity of SRP-associated complexes for a distinct subset of translated mRNAs (Figure 2B), consistent with the SRP specificity for a distinct subset of nascent polypeptides (Figure 2B, yellow highlight). Secretory pathway proteins (Figure 2C, yellow bars) were disproportionately represented among SRP-associated mRNAs, whereas the mRNAs encoding cytosolic and mitochondrial proteins (Figure 2C, cyan bars) were significantly under-represented among SRP-associated mRNAs. The consistency of these results with the known function of SRP suggests that this procedure can selectively identify the mRNAs encoding nascent polypeptides that are in vivo substrates of specific cotranslational chaperones.

The recognition code for SRP derived from in vitro studies provided a basis for several algorithms that predict SS and TM domains from sequence information; these are used to identify putative secretory pathway proteins (reviewed in [25]). The systematic identification of SRP substrates provides an unprecedented opportunity to benchmark these predictive algorithms against the experimentally determined SRP substrates from yeast. We used published algorithms (SignalIP, RPSP, TMHMM, HMMT, and the curated Uniprot database) to identify putative SS or TM regions encoded by mRNAs that were associated with SRP with high confidence (1% FDR) (Figure 2D and Figure S2A) as well as in the mRNAs least enriched in our SRP IPs (herein the “non-SRP interactors”) (Figure S2Bi and S2Bii). All these programs predicted, with good agreement, TM domains in ∼60%–75% of the SRP interactors (Figures 2D and Figure S2Aii, hairline denotes consensus among programs) and an SS in 15%–35% of the SRP interactors (Figure S2Aii). Notably, however, the algorithms found no SS or TM domains in about a quarter of the proteins encoded by SRP-associated mRNAs (Figure 2D, 102 targets when using SignalIP and TMHMM). These could represent *bona fide* SRP substrates that are recognized by novel, yet-to-be-determined features. Indeed, 12% of these proteins are annotated as membrane or secretory pathways (Figure 2E; Table 1). For instance, Sed4, a known integral ER membrane protein, and Sec20, a v-SNARE membrane glycoprotein involved in Golgi to the ER retrograde transport, are both encoded by mRNAs that we found to be enriched in association with SRP, though both lack predicted TM or SS regions. Despite the overall consistency of our results, some of the apparent interactions might be stochastic or spurious: For instance, 48% of proteins encoded by SRP-associated mRNAs that lack predicted TM or SS domains localize to the nucleus or mitochondrion (Table 1; Figure 2E). Future studies on the mechanistic and physiological significance of these potential non-canonical SRP interactors may reveal novel aspects of SRP function.

**Table 1:** SRP targets lacking predicted SS or TM.

GO category	Frequency	Genes
Unknown	63 (29.6%)	YAR030C, MOH1, YBL081W, YBR206W, YBR225W, MXR2, YCR015C, YCR025C, YCR102W-A, YDL023C, YDL034W, BSC1, YDR015C, YDR124W, YDR220C, PPM1, YEL076C, JHD1, YER137C, YER187W, THI5, ROG3, STR3, YGL199C, YGR018C, YGR066C, YHL049C, CRG1, YHR210C, YIL082W, YIR044C, YJL015C, YJL135W, YJL195C, YJL211C, HUL4, YJR087W, YJR107W, YJR146W, YKL153W, YLR236C, YLR255C, YLR463C, BSC3, YML002W, AIM32, YMR013W-A, YMR147W, YMR294W-A, YNL043C, YNL057W, YNL193W, YNL276C, BDS1, YOR021C, YOR041C, YOR093C, YOR203W, YOR248W, CIN1, YPL113C, YPL114W, YPR195C
Nucleus	61 (28.6%)	SWD1, CDC27, TKL2, UMP1, TDP1, THI2, SHG1, MAL33, THI3, DUN1, BPL1, MSH5, GAL3, DAD1, YDR132C, RPA14, SCC2, PRP42, IPK1, GCN4, NUG1, SPC25, HAC1, ACT1, RPL28, RTG, ZPR1, MAL13, SHU1, SRB2, BCY1, MSL1, TAD2, ZAP1, UTP18, PSF2, YJR008W, YJR027W, NMD5, TTI1, RGT1, PHD1, NUP120, MSN4, PMU1, POM34, CHA4, GSP1, GLO1, NSE5, NAT4, CEP3, UBP8, CSL4, SGO1, GSP2, RDR1, CTF19, REC8, YBL005W-A, YGR109W-A
Mitochondrion	31 (14.6%)	TIM12, YMC2, COS111, PGS1, CIT2, SLM3, GDH2, PTP1, YDR115W, RSM24, ACN9, PAD1, AGX1, ENO1, RRF1, ENO2, TAO3, TES1, OPI3, TTI1, YKL070W, GPM1, YKT6, CBT1, ALT1, MSS1, IRA2, CAT5, MGE1, ALD6, GIP3
Ribosome	10 (4.7%)	RPS14A, YDR115W, RSM24, RPL29, RPL28, RPS27B, RPS14B, RPS9A, GIP3, RPL1A
Endoplasmic reticulum	9 (4.2%)	SED4, LCB2, SRP101, DPL1, YDR476C, SEC20, OPI3, ERG5, GIP3
Endomembrane system	9 (4.2%)	SED4, COP1, LCB2, SRP101, SEC20, APL1, NUP120, POM34, RET3
Plasma membrane	3 (1.4%)	ENO2, BCY1, APL1
Vacuole	3 (1.4%)	ENO1, ENO2, YKT6
Golgi apparatus	2 (0.9%)	COP1, RET3

SRP interactors were analyzed for the presence of predicted Signal Sequences (SS) or Transmembrane Regions (TM). 213 targets do not have predicted SS or TM. Assignment of the corresponding cellular location was made retrieving GO ontology (component) categories from SGD.

As expected, applying the same algorithms to the proteins encoded by mRNAs not associated with SRP yielded few proteins with predicted SS or TM regions (Figure S2B; “non-SRP interactome”). Approximately 6% of these proteins had a predicted SS and ∼6% had a predicted TM domain (Figure S2B, note slight variations among algorithms). Interestingly, some of these proteins are annotated as localizing to the plasma membrane (Fus1p) or ER (Ost4p) and might therefore represent weakly SRP-bound or SRP-independent secretory proteins. Others correspond to mitochondrial proteins, which are generally not recognized by SRP; although dual targeting of some polypeptides to the mitochondria and the ER has been reported [26]. Still others, such as ribosomal protein Rpl45, contain a predicted SS yet are clearly cytoplasmic proteins.

Messenger RNAs encoding proteins with predicted TM regions were generally more highly enriched by our SRP affinity isolation procedure than proteins with predicted SS (Figure 2F), suggesting that the interaction of the correspondent nascent polypeptide with SRP was stronger or more sustained. Since TM regions are generally more hydrophobic than SS, this is consistent with previous biochemical experiments indicating that proteins with more hydrophobic sequences have a higher dependency on SRP for efficient ER translocation [27]. SRP-binding substrates lacking predicted SS or TM domains were typically less enriched than those containing either predicted TM or SS domains, suggesting that their SRP-binding sequences may be weaker and thus not recognized by algorithms designed to find sequences that bind strongly to SRP. While the hydrophobicity of the SS or TM regions is clearly important for SRP interaction, we only found a very weak correlation between this parameter alone and SRP enrichment (Figure S3 and unpublished data).

The presence of canonical SRP-binding, ER-targeting sequences in proteins that did not appear cotranslationally associated with SRP and the apparent enrichment of nascent proteins with no SS or TM regions in association with SRP suggest that our understanding of SRP specificity in vivo is still incomplete and that SRP-binding might be influenced by additional cis- and transacting factors.

### Contribution of SRP to Overall mRNA Membrane Targeting

A number of important questions surround the mechanisms and functions of mRNA association with membranes (reviewed in [28]). SRP inactivation is not lethal to yeast [29]–[31], indicating that SRP is not the only route to membrane association. mRNA binding proteins known to localize to cellular membranes could provide additional mechanisms for targeting selected mRNAs to the ER [32]. Experimental evidence that many mRNAs encoding cytosolic proteins associate with membranes has led to a suggestion that a substantial fraction of all translation in eukaryotic cells occurs in association with membranes [33].

**Figure 3:**
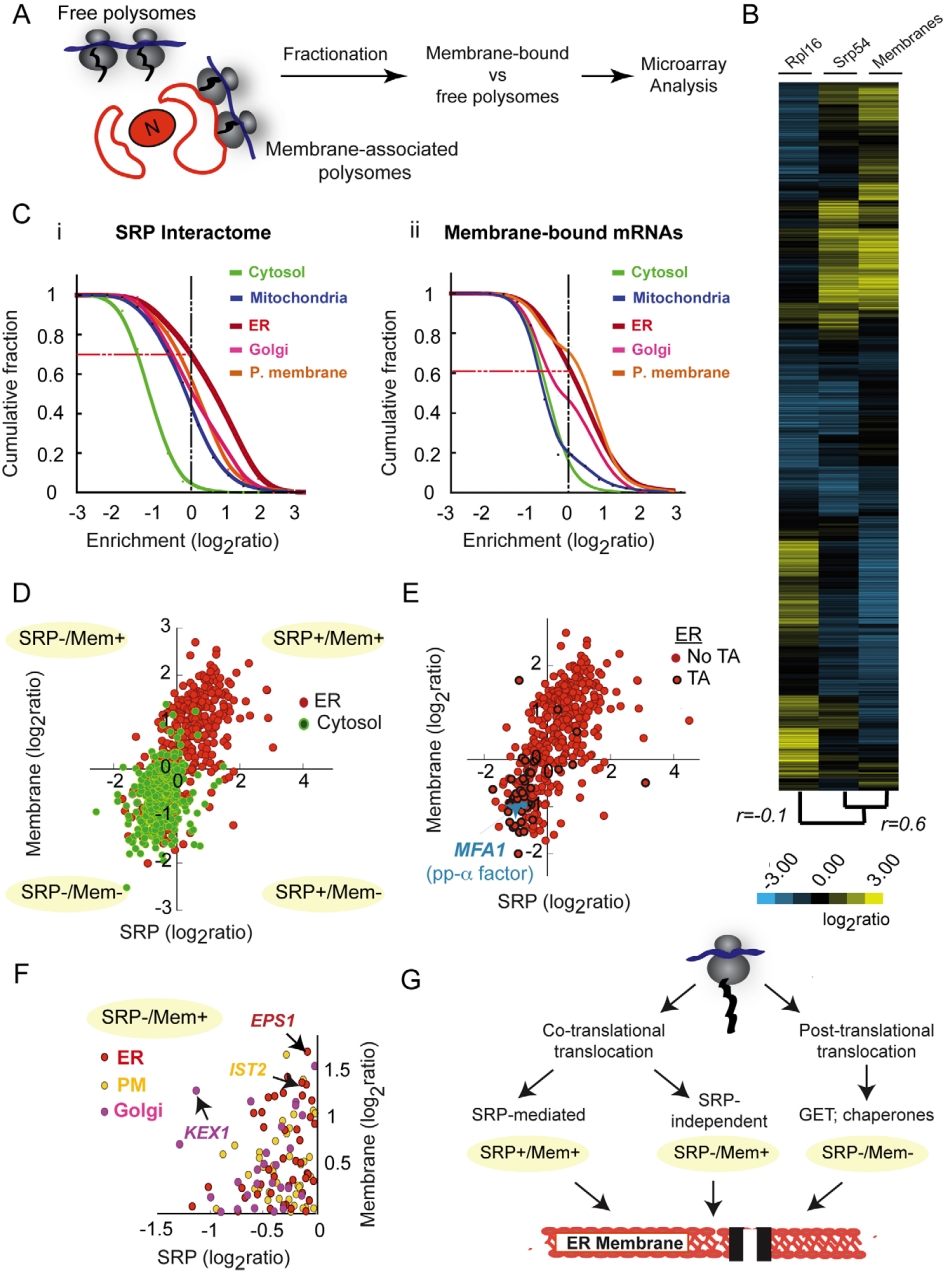
Global identification of membrane-associated mRNAs. (A) Experimental Strategy: Isolation of membrane-associated polysomes. Membrane-associated polysomes were separated from free polysomes by subcellular fractionation of WT yeast strains and associated RNA was isolated and then identified by microarray analysis. (B) Translational profile of SRP-associated and membrane-associated ribosomes. Hierarchically clustered heat map of the translation profiles obtained from immunopurifications made for TAP-tagged Srp54 and Rpl16 and from membrane-associated RNA. Each column represents the average of three different experiments and each row represents a gene. Pearson correlation coefficients between patterns of enrichment in the different immunoaffinity isolations determine the relationships represented by the dendogram as indicated. (C) Distribution of (i) SRP- and (ii) membrane-associated mRNAs encoding proteins of the indicated subcellular compartments. The graph represents the enrichment observed for mRNAs encoding proteins localized to various subcellular compartments as cumulative fraction of total mRNAs; 70% of ER proteins are enriched in the membrane associated fraction and 60% are enriched in association with SRP. (D) Scatterplot comparing the enrichment, represented as its log_2_ ratio, of individual mRNAs encoding cytosolic and ER proteins in the membrane fraction and in association with SRP. The indicated log_2_ ratio values are the average of three independent experiments. (E) Scatterplot comparing the enrichment of individual mRNAs encoding ER proteins in the membrane fraction and in association with SRP. mRNAs encoding Tail-anchored proteins (TA) are highlighted in black. Mfa1, a post-translationally translocated protein, is represented in blue. (F) Scatterplot highlighting mRNAs encoding secretory pathway proteins that were not enriched in association with SRP but were enriched in the membrane fraction. These are putative substrates of SRP-independent pathways of cotranslational translocation. (G) Pathways to the ER membrane. SRP- or membrane-associated pools can be compared to describe different targeting pathways. Cotranslational pathways require mRNA transport to the ER membrane (Mem+). Targeting to the ER membrane can be SRP mediated (SRP+/Mem+) or SRP independent (SRP−/Mem+). Posttranslational translocation pathways do not require mRNA transport to the ER membrane or SRP association (SRP−/Mem−) and are likely mediated by the GET pathway or by cytoplasmic chaperones.

To examine the contribution of the SRP-mediated route to overall mRNA targeting to membranes, we empirically defined the global complement of mRNAs associated with yeast membranes. We used a previously established differential centrifugation procedure [34],[35] to isolate membrane-associated mRNAs as well as the cytosolic, membrane-free mRNAs (Figure 3A). At a stringent statistical threshold (1% FDR), we identified 1,168 membrane-associated mRNAs (∼45% of the translatome, Figure 3B). Hierarchical clustering of SRP-bound and membrane-associated mRNAs demonstrated extensive overlap, as expected (Figure 3B; *r* = 0.6). A large fraction of membrane-bound mRNAs encoded proteins localized to ER, Golgi, or plasma membrane (Figure 3C i versus ii; red, pink, and orange, respectively), consistent with previous findings [35]. SRP-associated (Figure 3Ci) and membrane-associated (Figure 3Cii) fractions showed comparable enrichment for mRNAs encoding ER, Golgi, and Plasma membrane proteins. For instance, 60% of all mRNAs annotated as corresponding to ER proteins were enriched in the SRP-associated dataset (log_2_ ratio greater than 0) (Figure 3Ci, red line) and 70% were enriched in the membrane-bound dataset (log_2_ ratio greater than 0) (Figure 3Cii, red line). In contrast more than 90% of mRNAs encoding cytosolic proteins were included in neither the SRP-associated nor membrane-associated fractions (Figure 3C green). This result suggests that cellular membranes are not the major site of cytosolic protein synthesis, at least in yeast.

The enrichment for mRNAs encoding mitochondrial proteins was clearly higher in the membrane-associated than in the SRP-associated fractions (Figure 3C ii, blue line; Figure S4A and Table 2). This likely reflects the presence of mitochondria in our membrane preparation and supports the idea that a fraction of mitochondrial proteins are imported cotranslationally into mitochondria (reviewed in [36]).

**Table 2:** Selected proteins encoded by membrane-associated mRNAs that do not associate with SRP (Mem+/SRP−).

GO term	Frequency	Genes
Plasma membrane	(40) 7.4%	GPB2, APL3, FUI1, IST2, SUL1, PHO89, GIT1, GPR1, RGT2, SNF3, HXT15, DNF2, HKR1, HXT13, SHO1, RSP5, MSB2, MTL1, MAL11, DUR3, SLN1, PAN1, LSB6, HXT9, STE6, TRK2, FPS1, YPS3, PPZ1, DFG5, PLB3, YOL019W, SMF1, ALR1, HXT11, SLG1, NRT1, TRE1, OPY2, AQY1
Endoplasmic reticulum	(36) 6.7%	CNE1, SWH1, ALG14, SEC66, YPC1, ROT2, YDR056C, YOS9, GTB1, YEL043W, ERJ5, WSC4, EPS1, JEM1, MNS1, LHS1, SRP102, GPT2, MMM1, HRD3, UBX2, MSC1, ERO1, ASI1, SCJ1, LCB1, ASI3, PGA1, LRO1, ARE2, YNR021W, HRD1, MPD2, FLC1, ALG5, YPR091C
Endomembrane system	(27) 5%	CNE1, SWH1, APL3, NUP170, ALG14, SEC66, YOS9, NUP157, WSC4, NVJ1, EPS1, MPS3, JEM1, VPS35, SRP102, MMM1, HRD3, UBX2, NUP116, ASI1, LCB1, ASI3, PGA1, HRD1, SEC16, ALG5, APL4
Golgi apparatus	(13) 2.4%	SWH1, MNN2, SBE2, ANP1, RSP5, EMP47, KEX1, ATG27, MNN5, HOC1, KTR5, GNT1, APL4

Membrane-associated mRNAs and messengers enriched in SRP pulldowns were compared to generate a list of non-overlapping messengers. 541 total messengers were enriched in membrane and not enriched on SRP pulldowns. Assignment of the corresponding location was made retrieving GO ontology (component) categories from SGD. Only a select set of genes are listed here; categories not listed here include Mitochondrion (150 genes; 27.7%), Nucleus (69; 12.8%), Cell wall (22; 4.1%), Vacuole (24; 4.4%), and Unknown cellular component (179; 33.1%).

Joint analysis of the quantitative enrichment of each mRNA in association with SRP and membrane respectively gave further insight into modes of mRNA localization (Figure 3D–F and Figure S4). Comparison of both SRP and membrane-associated RNCs (significantly enriched targets at 1% FDR) reveals that most mRNAs that were both SRP-associated and membrane-associated (SRP+/Mem+) encoded proteins annotated as belonging to the secretory pathway (Figure 3D,E for ER; Figure S4B–D for Plasma membrane and Golgi). Interestingly, 24% of the SRP-associated RNCs in which the nascent polypeptide lacks either predicted SS or TM regions were also membrane-associated (Table S1); thus, these nascent chains are likely *bona fide* SRP targets despite their lack of a canonical SRP binding site. Virtually no transcripts encoding cytosolic proteins (Figure 3D, green) and few encoding mitochondrial proteins (Figure S3A; Figure S4C) were SRP+/Mem+. As expected, these mRNAs were overwhelmingly SRP−/Mem−. Notably, a number of mRNAs encoding secretory pathway proteins also fell into this class. We reasoned that these might represent proteins imported into the ER post-translationally. Indeed, known substrates of post-translational translocation pathways were SRP−/Mem− (Figure 3E). These include tail-anchored proteins (Figure 3E, TA, highlighted in black), which use the post-translational GET pathway [37] and pre-pro-alpha-factor (Mfa1, Figure 3E), which uses cytosolic chaperones to reach the ER membrane [38],[39]. Further analysis of this dataset may reveal additional substrates of these pathways.

Of particular interest were secretory pathway proteins whose mRNAs were membrane-associated but not SRP-associated (SRP−/Mem+; Figure 3F and Table 2), such as the chaperone *EPS1*, the plasma membrane protein *IST2*, and Golgi protease *KEX1*. These may represent translocation substrates whose mRNAs are targeted to membranes in an SRP-independent mechanism. Interestingly, *IST2* mRNA is known to localize to the bud tip by an actomyosin-driven process and is associated with cortical ER via an SRP-independent mechanism [40]. One possible mechanism for this process could be direct localization through specific membrane-associated RNA-binding proteins (RBPs) [32],[41]. However, we could not detect significant overlap between the SRP−/Mem+ mRNAs and the mRNA targets of previously described membrane-associated RBPs (unpublished data). Thus, novel yet-to-be-determined pathways and factors may function to localize these mRNAs to membranes.

### Defining Cotranslational NAC Specificity by Analysis of Associated Ribosome-Nascent Polypeptide Complexes

**Figure 4:**
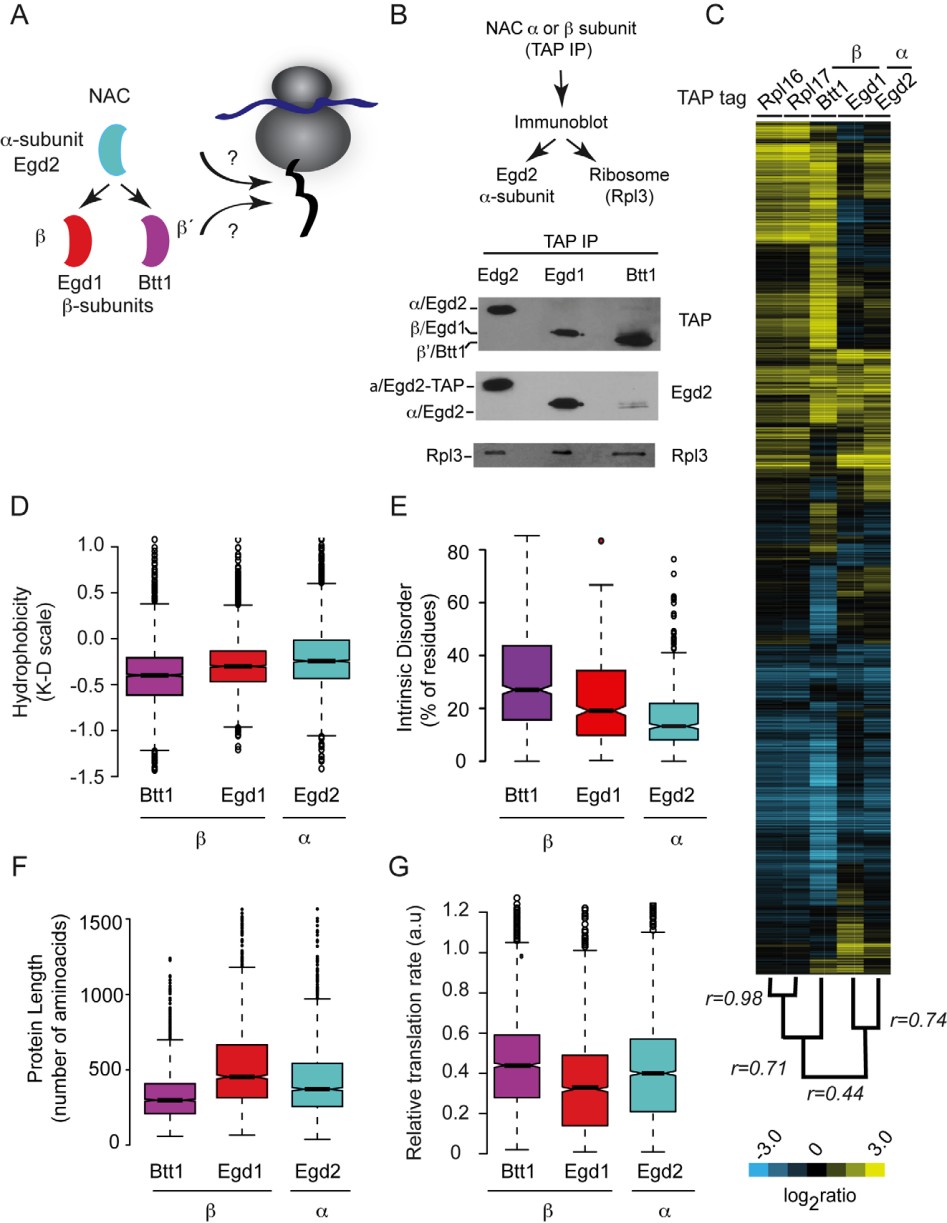
Global identification of polypeptides that interact cotranslationally with NAC. (A) Global approach to identify polypeptides that associate cotranslationally with NAC by affinity purification of α (Egd2) and β (Egd1 and Btt1) NAC subunits. (B) Immunoblot analysis of affinity purified NAC complexes. Association of the indicated NAC subunit with ribosomes was detected using anti-Rpl3 monoclonal antibody; Egd2 (α) subunit using anti-Egd2 polyclonal antibody and TAP-tagged proteins were detected with anti-TAP polyclonal antibody. (C) Hierarchically clustered heat map of the mRNAs associated with ribosomes and RNCs bound to the indicated NAC subunits. Each column represents the average of three replicates and each row represents a gene. Pearson coefficient correlations between experimental sets are indicated on the dendogram. (D–G) Physicochemical properties of polypeptides that associate cotranslationally with NAC. Box plots showing the distribution of the hydrophobicity (D), predicted intrinsic disorder (E), protein length (F), and relative translation rate (G) of NAC polypeptides cotranslationally associated with individual NAC subunits.

To gain insight into the cotranslational specificity of NAC, we systematically identified mRNAs cotranslationally associated with NAC complexes, using DNA microarrays to profile the mRNAs associated with each of the three NAC subunits, Egd2, Egd1, and Btt1 (Figure 4A). Importantly, dissociation of the ribosome-mRNA-nascent chain complexes with EDTA abrogated the association of NAC with mRNAs (unpublished data), suggesting that the mRNAs identified by this assay in association with individual NAC subunits reflect the cotranslational specificity of NAC for the nascent polypeptide.

Each of the TAP-tagged NAC subunits was ribosome associated (Figure 4B, lower panel; Figure 1B). The extent of α/β heterodimer formation for each β subunit was assessed by immunoblot analysis of α/Egd2 enriched by immunoaffinity purification of each of the two β isoforms (Figure 4B, middle panel, lanes 2 and 3). As expected, the *EGD1*-encoded β subunit was strongly associated with the α subunit, Egd2, consistent with previous reports [16],[42]. On the other hand, little of the Egd2/α subunit copurified with the *BTT1* encoded β' subunit (Figure 4B, compare lanes 2 and 3). This is consistent with evidence that Btt1 elutes predominantly at a homodimer molecular weight during size exclusion chromatography of yeast cell extracts [16].

Hierarchical clustering of the mRNAs based on their patterns of enrichment in association with each NAC subunit reveals two striking properties of NAC: First, there were clear differences between NAC subunits, suggesting that the different NAC subunits recognize different subsets of mRNA-RNC complexes. Second, NAC targets include virtually every mRNA associated with Rpl16/17, suggesting that at least one NAC isoform can interact with virtually every nascent polypeptide in the cell. This result is consistent with the estimated stoichiometry of NAC to ribosomes (1.25∶1) together with evidence that most of NAC in the cell are ribosome-bound [43]. Importantly, no mRNA was recovered by NAC complexes from non-ribosome-associated fractions (unpublished data), suggesting that the mRNA association and specificity are mediated through translating ribosomes (unpublished data). Similarly, omission of cycloheximide during cell extract preparation and analysis, which leads to polysome dissociation, dramatically reduced the number of mRNAs associated with Egd2 (Figure S5). Because association of Egd2 with mRNAs is critically dependent on the presence of intact polysomes, we conclude that Egd2 does not interact with mRNAs directly, but rather, through its association with translating nascent chains.

What determines the substrate specificity of different NAC subunits? The nascent proteins associated with different NAC subunits exhibited significant differences in a number of physicochemical properties, most notably length, hydrophobicity and intrinsic disorder, as well as inferred translation rate (Figure 4D–G). Btt1 associates with mRNAs encoding proteins of higher intrinsic disorder and lower hydrophobicity, whereas Egd2 associated with mRNAs encoding proteins with low intrinsic disorder and high hydrophobicity (Figure 4D,E). The length distribution of predicted protein products, which correlates inversely with the overall rate of folding (Figure 4F) [44], as well as translation rate of the mRNAs (Figure 4G) were also significantly different among sets of mRNA respectively associated with different NAC subunits. These differences suggest that each NAC subunit participates in recognizing specific features of the nascent polypeptide; Egd2 may have higher affinity for longer, more slowly folding polypeptides exposing hydrophobic determinants, whereas Btt1 may preferentially recognize more polar, disordered chains.

**Figure 5:**
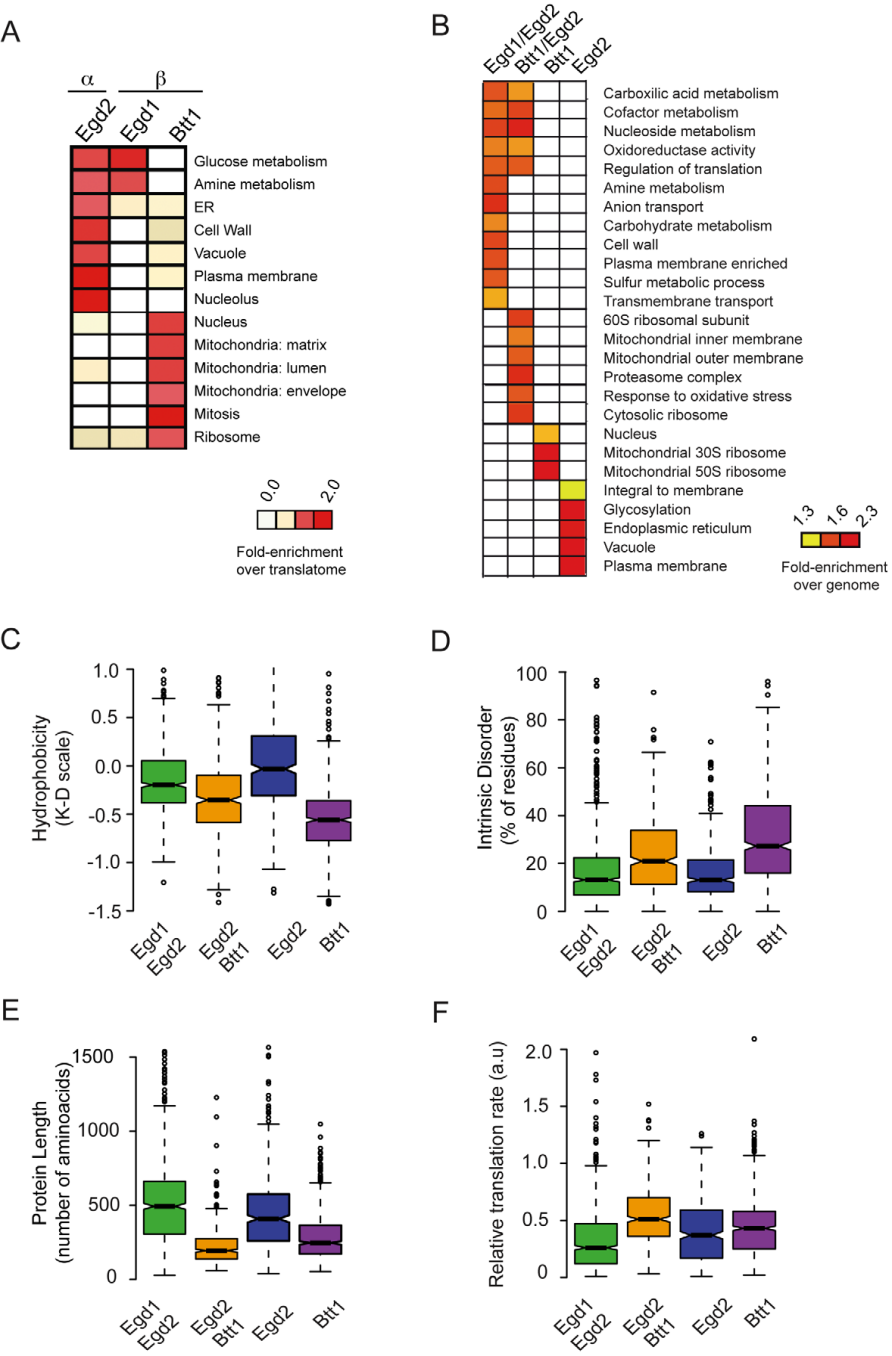
Distinct functional and physical properties of the polypeptides cotranslationally associated with NAC. (A) Heat map indicating GO categories (rows) differentially represented in the sets of polypeptides cotranslationally associated with different NAC subunits. Enrichment of a specific GO annotation is evaluated in comparison to the representation of that annotation in the proteins encoded by mRNAs significantly enriched in association with Rpl16. (B) Enrichment of different GO ontology terms (rows) in target sets (1% FDR) of different NAC heterodimers. For this analysis, nascent polypeptides associated with each of two NAC subunits were considered to be targets of the corresponding heterodimer. The significance of the enrichment of the GO term is represented as fold enrichment over the genome on a heat map. (C–F) Physicochemical properties of NAC cotranslational interactors classified as substrates of the indicated hetero- or homodimers. Box plots showing the distribution of the hydrophobicity (C), intrinsic disorder (D), protein length (E), and relative translation rate (F) of NAC cotranslational interactors considering potential NAC dimers. For this analysis, targets common to Egd1 and Egd2 or to Egd2 and Btt1, respectively, were considered to represent targets of the corresponding heterodimers; targets unique to Egd2 or Btt1 were considered to represent targets of the corresponding homodimers.

There were also differences in the distribution of functional roles of nascent chains associated with the different NAC (Figure 5A) subunits. Egd1 and Egd2 targets were enriched for mRNAs encoding metabolic enzymes, whereas the targets of Btt1 were enriched in mitochondrial and ribosomal proteins (Figure 5A). RNCs translating membrane and secretory pathway proteins were also associated with NAC α/Egd2. Preferential NAC association with nascent proteins sharing specific physicochemical properties may have resulted indirectly in the enrichment for specific functional categories. For instance, the preferential interaction with nascent ribosomal proteins with Btt1 may reflect its preference for short, highly disordered polypeptide chains with high translation rates. Alternatively, some features differentially associated with both overall physicochemical properties and functional roles of the translated proteins may underlie the observed differential specificity of NAC subunits.

Our GO analysis also revealed overlaps in specificity among pairs of subunits, most notably for Egd1 and Egd2. mRNA association patterns of these two subunits were similar to each other (*r* = 0.74 average of three replicates for every subunit) and more distinct from the alternative β subunit, Btt1 (Figure 4C). This is consistent with, and likely reflects, the predominance of the Egd1/Egd2 heterodimer in vivo [16]. NAC subunits appear to exist as a combination of homo- and hetero-dimers in the cell [10],[42], and each complex may have a different set of specificities. To explore this possibility, we extracted those substrate sets shared by a α/β pair: that is, likely Egd1/Egd2 or Btt1/Egd2 substrates, and those associating solely with individual NAC subunits, that is, likely substrates of a NAC homodimer. We thus examined whether specific functional themes were significantly enriched in each category (Figure 5B). Few nascent polypeptides associated with Egd1 alone, suggesting that Egd1 primarily functions in a complex with Egd2. Egd1/Egd2 preferentially associated with RNCs translating proteins that function in carbohydrate metabolism, while the Btt1 and Btt1/Egd2 preferentially associated with RNCs translating mitochondrial and ribosomal proteins (Figure 5B). Some protein classes, including redox and nucleotide metabolism, interacted with all NAC subunits, whereas Egd2 only and to a lesser extent Egd1/Egd2 also associated with RNCs translating secretory proteins; notably, this subset of nascent polypeptides also associated with SRP.

We next examined how this analysis reflected on the physicochemical properties of substrates (Figure 5C–F). Incorporating into our analysis the idea that NAC exists as heterodimers and homodimers exacerbated the differences in the intrinsic properties observed for each subunit set. The binding specificities of Egd2/Egd1 and Egd2/Btt1 appeared to reflect the combined specificity of the subunits in the dimer (Figure 5C–F; green, Egd1/Egd2; orange, Egd2/Btt1; blue, Egd2/Egd2; purple, Btt1/Btt1). In contrast, the nascent polypeptides associated exclusively with Btt1 (Figure 5C,D, purple) comprised proteins with the highest intrinsic disorder and lowest hydrophobicity, whereas the RNCs associated with Egd2 translated the most hydrophobic proteins (Figure 5C, D blue). Importantly, the fact that the interaction specificity of each subunit correlated so strongly with the predicted physical properties of the translated polypeptide is strong evidence that each NAC subunit recognizes determinants in the nascent chain itself. Furthermore, both components of each NAC dimer appear to contribute to nascent chain recognition, expanding both the specificity and number of RNCs recognized by NAC. Although different NAC homo- or heterodimers differentially associated with ribosomes translating different sets of mRNAs, the specificity of the ensemble of NAC complexes appears to encompass virtually every translated polypeptide.

### NAC Is a Modulator of SRP Specificity

**Figure 6:**
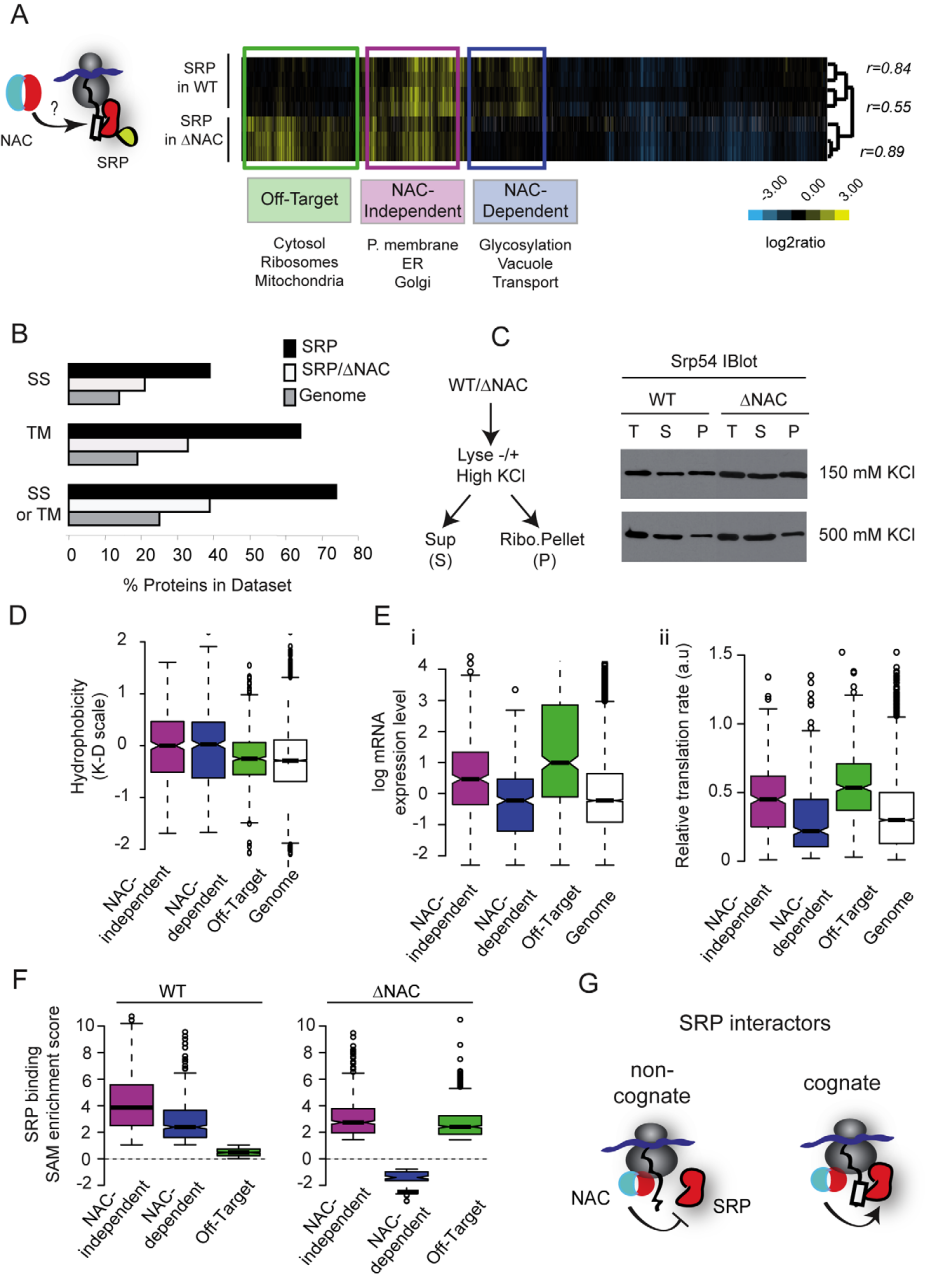
NAC is a global modulator of SRP specificity in vivo. (A) Hierarchically clustered heat map of the mRNAs cotranslationally associated with SRP in either the wild type (WT) or NAC-deleted (ΔNAC) cells. Boxes indicate genes significantly enriched (*p*<0.01) in association with SRP in both strains (purple) (NAC-Independent) or only in ΔNAC cells (green) (Off-target) or WT cells (blue) (NAC-Dependent). GO ontology categories significantly enriched (*p*<0.01) in each data set are indicated. Pearson coefficient correlations are indicated on the dendrogram. (B) Comparison of fraction of SRP substrates with predicted signal sequences (from Signal IP) or transmembrane regions (from TMHMM) in WT cells (black), ΔNAC cells (white), or the yeast genome (grey). (C) SRP association with ribosomes is unchanged by deletion of NAC. Total yeast lysates (T) were separated into ribosomal (P) and non-ribosomal fractions (S) through sedimentation on sucrose cushions at different salt concentrations (150 and 500 mM). SRP presence in the different fractions was determined by western blot using a polyclonal anti-TAP antibody. (D) Box plots representing the N-terminal hydrophobicity of different SRP cotranslational interactors: NAC-dependent, NAC-independent, and Off-target SRP interactors compared to the genome. (E) Distribution of mRNA expression level (i) and translation rate (ii) for different classes of SRP associated mRNAs. (F) Box plots representing the distribution of enrichment values (SAM score) for NAC-dependent, NAC-independent, and Off-target SRP interactors in WT and ΔNAC cells. (G) Schematic representation of SRP modulation by NAC. NAC favors SRP binding to cognate interactors (ER-destined proteins) and prevents binding to non-cognate interactors (cytosolic proteins).

The role of NAC in SRP specificity and substrate selection has been a matter of debate [17],[45],[46]. NAC and SRP both contact the ribosomal protein Rpl25 [3]. NAC was originally proposed to compete with SRP for ribosome binding [17]. However, our global analysis revealed that many nascent secretory pathway proteins can interact with both SRP and the NAC subunits Egd2 and Egd1. We tested whether the specificity overlap might reflect joint binding at the ribosome. We isolated SRP-containing RNCs and tested for the presence of NAC (Figure S6A). Indeed, immunopurification via either Srp54p, Srp68p, or Srp72p revealed the presence of Egd2p in SRP-associated polysomes (Figure S6A), suggesting that NAC and SRP might bind simultaneously to the same RNCs, though this experiment does not exclude that these factors might bind to different ribosomes engaged in translation of the same mRNA. However, NAC does not detectably affect the extent of SRP association with ribosomes, as shown by the similarity of SRP cofractionation with polysomes in WT and *Δegd1Δegd2* cells (, see also below Figure 6B).

To further explore the functional interplay between SRP and NAC, we examined whether the absence of NAC affects SRP recognition of nascent proteins, as reflected by its pattern of association with mRNA (Figure 6). To this end, we compared the ribosome-nascent polypeptide interaction specificity of SRP in wild type cells (herein NAC+) with that in cells lacking NAC subunits Egd1 and Egd2 (*Δegd1Δegd2*, herein ΔNAC) (Figure 6A). Clustering analysis highlights the striking differences between the patterns of SRP-bound mRNAs in NAC+ and ΔNAC cells (Figure 6A; Figure S6A). SRP association with a core set of RNCs encoding secretory proteins was relatively independent of NAC, as these were enriched by SRP affinity isolation in both NAC+ and ΔNAC cells (Figures 6A and S7A, purple). In contrast, the SRP-association with another set of secretory proteins appeared to be NAC-dependent, as it was lost in ΔNAC cells (Figures 6A and S7A, blue). Two SRP-dependent proteins, DPAPp and Kar2p [27], fell into this category (Figure S7A). Strikingly, a third set of mRNA-RNC complexes only associated with SRP in ΔNAC cells; most of these mRNAs encoded cytosolic proteins (Figures 6A and S7A, green; Off-target interactors). The relative depletion from the SRP-associated RNCs of transcripts encoding “bona fide” SRP substrates, that is, secretory pathway proteins and the corresponding increase in mRNAs encoding cytosolic proteins from the SRP-associated RNCs, was also reflected in the GO analysis of the proteins interacting cotranslationally with SRP (Figure S7B). Using a 1% FDR to analyze the SRP interactomes, we find that 70% of SRP-associated RNCs in NAC+ cells contained nascent polypeptides with predicted SS or TM regions (Figure 6B) whereas in ΔNAC cells, only 40% of the SRP-associated mRNAs encoded proteins with SS or TM domains. Of note, the depletion in “bona fide” SRP substrates in ΔNAC cells was independent of the statistical stringency of the analyses (Figure S8).

Interestingly, deletion of the second NAC isoform (*Δegd2/Δbtt1*) (ΔNAC') had a similar effect on SRP specificity (Figure S8). As observed for ΔNAC cells, the SRP interactome in ΔNAC' cells was also depleted in proteins containing predicted SS or TM regions (Figure S8B, Figure S8C) and mRNAs encoding secretory proteins (Figure S8D). In these cells, SRP also associated with more cytosolic and mitochondrial proteins (Off target, Figure S8A, Figure S8D). Thus, NAC significantly influences the in vivo specificity of SRP interactions.

How does NAC affect SRP specificity? The extent of SRP interaction with ribosomes and the salt-sensitivity of this interaction were not affected by the absence of NAC (Figure 6C). The fact that NAC appears to enhance the association of SRP with some nascent polypeptides (i.e., NAC-dependent) and prevent SRP interactions with others, leaving still others unaffected, suggests a complex mode of regulation. We first hypothesized that less hydrophobic SRP-binding nascent polypeptide sequences might be more easily displaced in the absence of NAC. Our analysis did not support this hypothesis; NAC-dependent and NAC-independent SRP interacting proteins were indistinguishable based on the length and hydrophobicity of their predicted SS or TM domains (Figures 6D, S7C and unpublished data). mRNA abundance and translation rate provided the strongest identifiable differences between NAC-dependent and NAC-independent SRP interactions (Figure 6E, note log scale in 6Ei). NAC-independent SRP substrates were relatively highly translated, abundant proteins; NAC-dependent SRP substrates tended to be much less abundant membrane and secreted proteins (Figure 6E). Because abundance and translation rate appeared key to the NAC-modulation of SRP specificity, we compared the relative enrichment of each class of SRP-associated mRNAs in the presence or absence of NAC (Figure 6F). The abundant, NAC-independent SRP substrates were the most highly enriched SRP interactors even in wild type cells; their level of enrichment was only modestly affected by the absence of NAC. In contrast, the NAC-dependent SRP substrates were less highly enriched in association with SRP, even in wild type cells. Their interaction with SRP was completely undetectable in the absence of NAC. The nascent cytosolic nascent proteins whose latent ability to interact with SRP was apparently blocked by NAC were generally highly abundant cytosolic proteins with high translation rates (Figure 6E, Off-target). These cytosolic proteins do not bind appreciably to SRP in wild type cells, but displayed an enrichment level comparable to *bona fide* SRP substrates in the absence of NAC, despite their lack of canonical SRP binding sequences.

It is known that SRP can recognize hydrophobic sequences with broad specificity [47]. One of the most striking aspects of SRP function observed here is that, in vivo, in wild type cells, SRP displays exquisite specificity for its cognate substrates independent of their concentration in the cell. In the absence of NAC, however, SRP also interacts with very abundant nascent polypeptides that lack high affinity SS or TM binding sites. These may compete for SRP, effectively lowering its availability to sample less abundant cognate sequences. NAC thus effectively acts as both a positive and negative regulator of SRP interactions with potential binding targets, tuning out the noise and enhancing the specific interactions with low abundance cognate substrates (Figure 6G).

### Robustness of Membrane Targeting Pathways in the Absence of NAC

**Figure 7:**
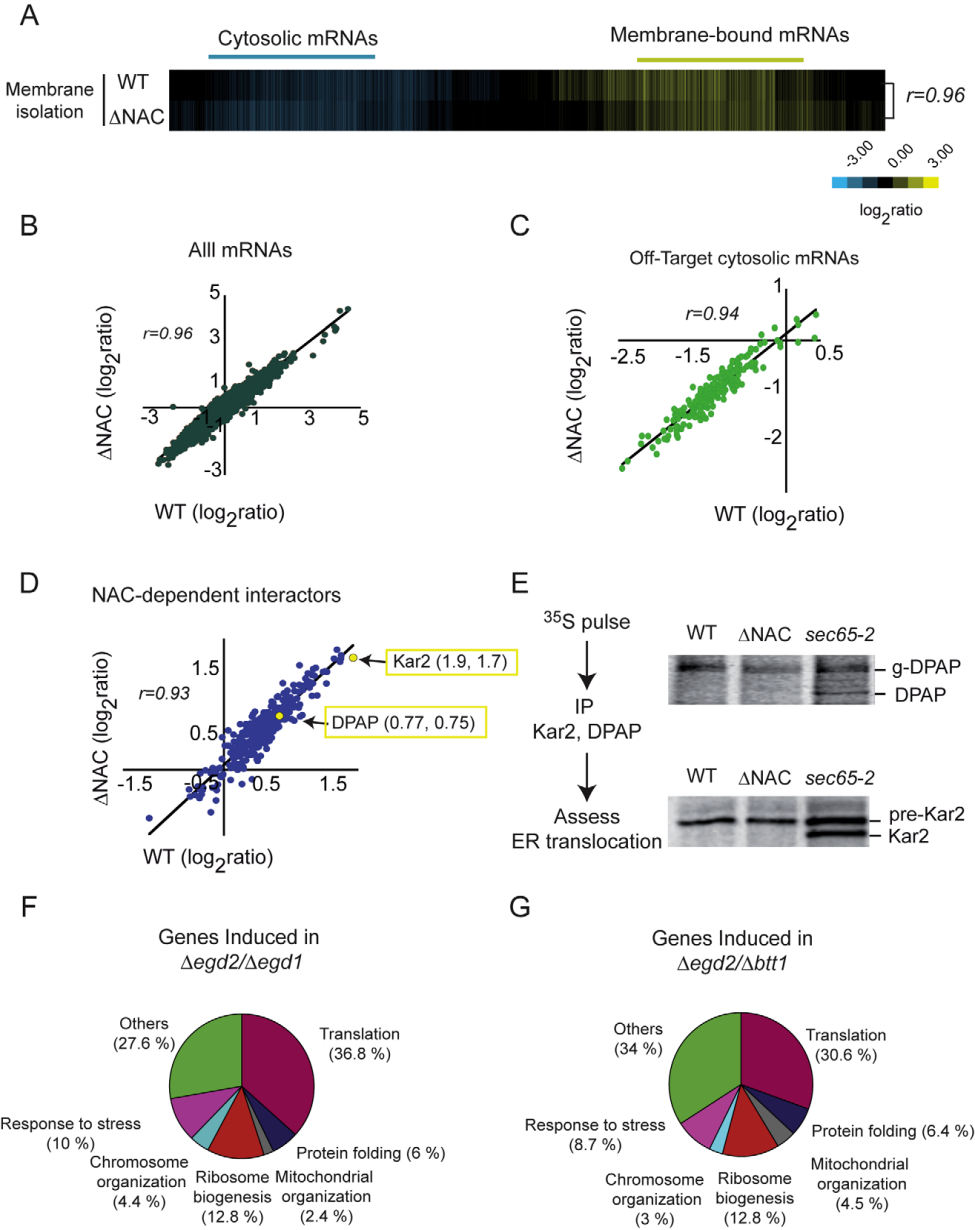
Robustness of protein homeostasis and translocation compensates for NAC deletion. (A) Hierarchically clustered heat map of mRNAs in membrane-associated RNCs in WT and ΔNAC cells. (B) Scatterplot comparing the enrichment of individual membrane-associated mRNAs in wild type and ΔNAC strains. All points fall in a diagonal line, indicating that loss of NAC does not impair membrane targeting. (C) Scatterplot comparing the membrane enrichment for mRNAs encoding cytosolic Off-target SRP interactors in WT and ΔNAC strains. No increase is observed for the ΔNAC cells. (D) Scatterplot comparing the membrane enrichment for mRNAs encoding NAC-dependent SRP interactors in WT and ΔNAC strains for membrane-associated mRNAs. Values obtained for two known SRP targets, Kar2 and DPAP-B, are highlighted. (E) Translocation of Kar2 and DPAP-B is not affected by deletion of NAC. WT, ΔNAC, and *Sec65-1* cells were pulse-labeled with ^35^S-methionine, immunoprecipitated with specific α-Kar2 and α-DPAP antibodies, and analyzed by SDS-PAGE and phosphorimaging. Positions of mature cleaved Kar2 (Kar2) and glycosylated DPAP (g-DPAP) and its precursors (uncleaved pre-Kar2 and DPAP) are indicated. (F–G) Transcriptional response to deletion of NAC. Pie charts show the distribution of genes upregulated in *Δegd2/Δegd1* (F; 299 at 1% FDR) and *Δegd2/Δbtt1* (G; 310 at 1% FDR).

Deletion of NAC has few phenotypic consequences for the cell (unpublished data) [16],[21], while loss of SRP function severely compromises growth. We reasoned that if SRP were to bind inappropriately to nascent cytosolic proteins in the absence of NAC and directs the paused ribosome to the ER, the associated mRNA should inappropriately localize at the ER membrane. We thus investigated whether loss of NAC would also affect global mRNA targeting to the ER (Figure 7A). Strikingly, the distribution of mRNAs between the membrane-associated and soluble fractions was indistinguishable between NAC+ and ΔNAC yeast cells (Figure 7B; *r* = 0.96). For instance, cytosolic proteins that inappropriately interacted with SRP in ΔNAC cells (Figure 7C, “Off-target”, green) were nevertheless largely absent from the membrane fraction in both wild type and ΔNAC cells (Figure 7C). Conversely, the “NAC-dependent” nascent polypeptides whose association with SRP was impaired in ΔNAC cells still efficiently associated with membranes in ΔNAC cells, despite their diminished association with SRP (Figure 7D, blue, compare with Figure S7B). We conclude that despite the loss in SRP specificity under these conditions, loss of NAC activity has little or no effect on the fidelity of mRNA targeting to membranes, despite the loss in SRP specificity.

We chose two NAC-dependent SRP substrates, Kar2 and DPAP, whose association with SRP but not with membranes was impaired in ΔNAC cells, for further biochemical analysis (Figure 7E). The efficiency of their ER translocation in wild type or ΔNAC cells was evaluated by determining the ratio of processed versus unprocessed protein following a short pulse with ^35^S-methionine. Defective translocation results in accumulation of precursors of these two proteins, that is, uncleaved Kar2 (pre-Kar2) and non-glycosylated DPAP. The presence or absence of NAC did not affect the speed or efficiency of translocation for either Kar2 or DPAP (Figure 7E). In contrast, impairing SRP function using the temperature-sensitive mutation *sec65-2* did reduce translocation of both proteins. Thus, the apparent decrease in SRP binding of these SRP substrates in the absence of NAC did not appreciably impair their translocation to the ER. These experiments highlight the robustness and fidelity of membrane targeting pathways.

To better understand how the cell compensates for the loss of NAC function, we examined the transcriptional response to joint deletion of either Egd1 and Egd2 or Egd2 and Btt1 (Figure 7F). A different complement of genes was induced in response to these two perturbations, but major features of the responses were shared. Transcripts encoding ribosomal proteins, ribosome biogenesis and mitochondrial biogenesis machines, and chaperones and stress response genes were induced in response to both defects (Figure 7F and Tables 3 and 4). Notably, loss of NAC activity did not lead to transcriptional induction of an unfolded protein response (UPR), consistent with the lack of a translocation defect in these cells (Figure 7F and unpublished data). The chaperones induced in response to NAC deletion included stress-inducible chaperones like *SSA3* and small HSPs, as well as CLIPS, most notably *SSB1/2*. The synthetic genetic interaction between *SSB1/2* and the NAC complex suggests that induction of *SSB1/2* may contribute to functional compensation for the loss of NAC [21]. The induction of ribosomal proteins and ribosome biogenesis genes is in accord with the observation that NAC has a role in ribosome biogenesis ([21]; Figures 4 and 5). Loss of Egd2/Btt1 led to induction of numerous mitochondrial biogenesis factors, including *AFG3*, *SED1*, and *MIA4*, suggesting a role for this NAC complex in mitochondrial biogenesis.

**Table 3:** Selected genes upregulated in *Δegd2/Δegd1* cells.

GO term	Frequency	Genes
Protein folding	6%	SSB1, AHA1, SSA4, SSA2, HSP104, CPR6, HSP60, TSA1, HSC82, SIS1, SSB2, HCH1, STI1, SSE1, HSP82
Ribosome biogenesis	37%	RPS8A, RPS14A, NOP1, RPS16B, RPL35B, RPL35A, SSB1, RPS3, RPL12B, RPS17B, RPS18A, RPL12A, RPS24A, RPS26B, RPL30, RPS2, RPS26A, RPL11B, RPS23A, RPS27B, RPS24B, RPL40A, RPS21B, RPS5, RPS21A, RPS0B, RPL10, RPS31, RPP0, REH1, RPL6B, RPS17A, RPS18B, RPL6A, RPS16A, RPS3, SSB2, RPS19B, RPS15, RPL25, RPS28A, RPL5
Mitochondrion organization	2.4%	SED1, AFG3, HSP60, HSP82, YME1, YLH47

Only a select subset of upregulated genes are listed here, GO ontology categories (Process) not listed include Translation (92; 36.8%), RNA metabolic process (35; 14%), and Response to stress (25; 25%).

**Table 4:** Selected genes upregulated in *Δegd2/Δbtt1* cells.

GO Term	Frequency	Genes
Protein folding	6.4%	FLC2, SSB1, AHA1, SSA4, SSA2, HSP104, CPR6, HSP60, TSA1, HSC82, SIS1, YDJ1, SSB2, HCH1, STI1, SSE1, HSP82
Ribosome biogenesis	12.8%	RPS14A, RPL35B, RPL35A, SSB1, RPS13, RPL12B, SNU13, RPL12A, RPS24A, RPL30, RPS2, RPS26A, RPL11B, RPS23A, RPS24B, RPL40A, RPS21B, RPS5, RPS21A, RPL40B, RPS0B, RPL10, RPS31, RPP0, RPS18B, RPL6A, RPS16A, RPS7B, RPS3, SSB2, RPS19B, RPS15, RPS19A, RPL5
Mitochondrion organization	4.5%	VAR1, SED1, AFG3, RPN11, MIA40, MEF1, HSP60, YTA12, YDJ1, HSP82, YME1, YLH47

Only a selected set of genes are listed here, GO ontology categories (Process) not listed include Translation (81; 30.5%) and Protein metabolic process (115; 43.4%).

## Discussion

### A Global Approach to Probe the Cotranslational De Novo Folding Network

Affinity isolation of cotranslationally acting chaperones from cells under conditions that preserve their interaction with the nascent polypeptide and associated ribosomes and quantitative profiling of the associated mRNAs open a window on the specificity and interplay of chaperones and targeting factors responsible for cotranslational protein homeostasis. This approach should enable us to probe the structure of the CLIPS network and the interplay between different chaperones and targeting systems. Unlike previous studies defining chaperone interactors by proteomic analysis, our approach focuses on cotranslational interactors as potential chaperone substrates. The approach presented here opens a window to understand the pathways and principles of cotranslational chaperone action.

### SRP Specificity and Cotranslational Targeting of Proteins to the ER

The full complement of nascent chains that interact with SRP in vivo has never been defined. Studies of SRP recognition using model substrates and peptides have shown that SRP recognizes highly hydrophobic signal sequences and transmembrane regions [27] but can also recognize hydrophobic stretches found in cytoplasmic proteins [39],[48]. We find that in vivo SRP displays considerable specificity for previously defined recognition sequences; approximately 80% of the in vivo substrates we identified contained a predicted SS or TM domain (Figure 2D). Our analysis also indicates that additional factors contribute to SRP specificity and affinity in vivo: approximately 20% of SRP interactors lack a discernible SS or TM domain. Since several SRP-associated mRNAs encode secreted or membrane-associated proteins that appear to lack canonical SS or TM domains, these interactions may be functionally relevant. Conversely, a number of proteins with clear SS or TM domains were translated in these cells but were not enriched in association with SRP, suggesting that SRP recognition might be regulated by features or mechanisms beyond its intrinsic affinity for SS or TM regions. Interestingly, it has been reported that, in bacteria, basic residues promote binding of SRP to a subset of signal peptides whose hydrophobicity falls slightly below a critical level [49]. Recent studies also suggest that a hydrophobic stretch can recruit SRP to the ribosome before it emerges from the ribosomal exit tunnel, presumably by changing the conformation of the ribosome [50],[51]. SRP-substrates without canonical SS or TMs may contain sequence elements that similarly enhance binding to SRP by this indirect mechanism or even by recruiting bridging factors.

The SRP-interactome also yielded some surprising observations. Although the glucose metabolism enzyme glyceraldehyde-3-phosphate dehydrogenase (GAPDH, Tdh1-3 in yeast) is reportedly mostly cytoplasmic [52], the mRNAs encoding all Tdh isoforms (i.e., Tdh1-3) were both SRP-associated and enriched in the membrane fraction (see Table 2). Notably, all three isoforms of this enzyme in yeast have predicted signal sequences at the N-terminus and have been detected on the outer surface of the cell wall [53]. Our findings suggest a mechanism by which Tdh can reach the outer cell wall and may also explain the observation that other primarily cytosolic proteins, including glycolytic enzymes, are secreted by yeast spheroplasts and found as integral components of the cell wall [54],. Interestingly, GAPDH mRNA has also been found to associate with membranes in mammalian cells [56].

The variable efficiency of the SRP-export pathways for different mRNAs has been recognized both as a regulatory mechanism and as a source of misfolded proteins [57]. Even a small fraction of untranslocated precursors may represent a substantial burden for the cytosolic protein quality control machinery. Our analyses show quantitative variation in binding of SRP to its targets, which may be related to previous observations that some secretory and membrane proteins are more efficiently translocated than others [58],[59]. Differential translocation efficiency is proposed to underlie disease pathologies, such as prion formation, and to regulate protein flux into the secretory pathway [57],[60]. We find that mRNAs encoding proteins with predicted TM regions are more enriched in association with SRP than either polypeptides with SS domains or those with no detectable SS or TM domains (Figure 2F). Notably, in bacteria, SRP is a main targeting factor for membrane proteins, while secretory proteins follow a different, SecB-dependent, pathway (reviewed in [61]). Although differences among signal sequences have been shown to modulate translocation in yeast for a small number of substrates (reviewed in [57],[62]), we could not identify a clear correlation between SRP enrichment and any defined physicochemical property in the SS or TM domains themselves, such as length, overall or maximal hydrophobicity, or amino acid composition. In bacteria, the codon adaptation index of the SS and the efficiency in translation initiation have been proposed to influence targeting efficiency (reviewed in [63]); we found no significant correlation between SRP enrichment and these parameters (unpublished data). Our data indicate that, in vivo, the features that control SRP recognition of a given nascent polypeptide are more complex than expected. In principle, additional features promoting a pre-recruitment of SRP to translating ribosomes could increase the efficiency of SRP-dependent translocation and enhance physiological robustness. For example, the translation properties of a given mRNA might influence the efficiency with which a potential SS or TM domain or other hydrophobic stretches are recognized. Additional ribosome-associated factors could also modulate the SRP association with nascent chains, as shown here for NAC. What determines the variable efficiency we observe in SRP association with SS and TM containing nascent polypeptides remains an important unanswered question.

### Beyond SRP: Alternative Targeting Pathways to the ER Membrane

The comparative analysis of SRP- and membrane-bound mRNAs provides an overview of overall partitioning of co- and posttranslational events in membrane targeting. Our data indicate that most cytosolic mRNAs are not membrane-associated, suggesting the existence of mechanisms that separate cytosol-bound from membrane-bound mRNAs. SRP appears to be involved in cotranslational targeting of most membrane and secretory proteins to the ER; ∼80% of membrane-associated mRNAs encoding these proteins were also SRP-associated, at a 1% FDR threshold. However, we also found evidence for SRP-independent translocation pathways. A significant minority of mRNAs, roughly 20%, appears to associate with membranes through SRP-independent pathways. We estimate that 25% of the secretory pathway proteins that do not associate cotranslationally with membranes are translocated posttranslationally to the ER; these include tail-anchored proteins, which use the GET pathway [37],[64], and Mfa1, whose translocation is assisted by cytosolic chaperones including Hsp70 and TRiC/CCT [38],[39].

SRP is not essential in yeast [65] and downregulation of SRP in mammalian cells has a mild effect on growth [33],[66], indicating the existence of SRP-independent mechanisms for translocation [27]. The membrane-associated mRNAs that encode membrane or secreted proteins but do not bind SRP (SRP−/Mem+) are candidates for substrates of SRP-independent cotranslational translocation pathways. Little is known about these pathways. They may involve direct recruitment of RNCs to the Sec61 translocon [67], as well as additional factors [31],[68]. Direct, translation-independent targeting of mRNAs to membranes could also involve RNA-binding proteins (RBPs) such as Puf1, Puf2, Pub1, Scp160, Ypl184c, Khd1 [41], and Whi3 [69], all of which bind distinct sets of mRNAs encoding membrane or secreted proteins. Interestingly, while few of these RBPs bind mRNAs in the SRP−/Mem+ set, there is also considerable overlap between their targets and the mRNAs we found enriched in association with SRP (unpublished data), suggesting these RBPs may provide redundancy or another level of control to cotranslational SRP targeting to membranes. Our experiments will provide an opportunity to refine our understanding of the signals and features that direct secretory proteins along these alternative non-SRP pathways.

The functions and localization patterns of mRNAs that were membrane-associated but not SRP-bound suggest several additional roles for SRP-independent membrane sorting of mRNAs. Many of these (150 out of 541) encoded mitochondrial protein precursors, which may be imported cotranslationally into mitochondria [70]. Among the remaining non-mitochondrial mRNAs, there was a paucity of mRNAs encoding ER-localized proteins (Lro1) and proteins with transmembrane domains, but the set included many mRNAs encoding proteins involved in other membrane systems in the cell. Two other She2 targets, Mtl1 and Lsb1, were included in this set and may also be associated with the cortical ER during trafficking to the bud [71]. Most strikingly, there were a number of mRNAs encoding proteins involved in endocytosis and actin patch assembly (Vps35, Aly2, Swh1, Lsb6, and Pan1), clathrin-mediated vesicle transport (Apl4, Apl3, Laa1, and Sec16), bud formation (Sbe2, Ypk1, Lrg1, Prm10, and Bem3), and vacuole function and assembly (Sch9, Vps13, Vac8, Tre2, Tre1, Fab1, and YIR014W). Potentially, these mRNAs are localized to specific membrane compartments to preferentially translate the proteins near their site of action. Alternatively, the nascent polypeptides could associate cotranslationally with membrane-associated interacting partners.

The set of membrane-associated mRNAs included an abundance of regulatory factors, including transcription factors (Stp2, Ino2, and Ppr1), RBPs (Puf2 and Puf3), and signaling molecules (Tor1, Bem3, Fab1, and Sch9). Localization of these mRNAs to appropriate membrane structures may facilitate co-translational association of their products with localized signaling partners or enable locally controlled regulation of their translation by signaling systems linked to these membranes. For instance, Stp2 promotes expression of permease genes and is synthesized as an inactive precursor that associates with the plasma membrane and is cleaved upon sensing of external amino acids [72]; Tor1, a component of the TOR complex, is a peripheral membrane protein that regulates cell growth in response to nutrient availability and stress [73], and Bem3 is a Rho GTPase activating protein specific to Cdc42, which controls establishment and maintenance of cell polarity, including bud-site assembly [74].

### Uncovering the Cotranslational Specificity of the NAC Complex

The abundant, ubiquitous, and evolutionarily conserved Nascent Chain Associated Complex (NAC) binds ribosomes in close proximity to the nascent chain exit site [75]. Despite its conservation, little is known about its function. Our analysis of the association of the three NAC subunits with nascent polypeptides revealed a surprising and unanticipated division of labor. Considered as a group, the three NAC subunits have translation-dependent interactions with almost every mRNA. Each subunit, however, exhibits distinct specificity for RNCs engaged in translation of mRNAs with different functional themes.

Based on the crystal structure of the archaeal NAC domain, NAC complexes are obligate dimers, where two subunits must complete the folded beta-sheet NAC-domain. Our analysis supports the idea that NAC subunits can function as either homodimers or heterodimers [10],[16]. We found a large overlap between the sets of transcripts associated with Egd1 and Egd2, consistent with the idea that the Egd1/Egd2 complex is the most abundant form. This dimer associated preferentially with nascent metabolic enzymes, including those in carbohydrate metabolism, such as glycolysis. Egd2, either as a homodimer or in a complex with Egd1, was also cotranslationally associated with a large fraction of mRNAs encoding membrane or secreted proteins, many of which also associate with SRP. Btt1, either as a homodimer or in a seemingly minor Btt1/Egd2 complex, associated primarily with RNCs translating ribosomal proteins and nuclear-encoded mitochondrial proteins.

In yeast, the three NAC subunits can be deleted with minimal impact on growth. Deletion of all three NAC subunits leads to enhanced ribosomal protein aggregation in cells also lacking the Hsp70 homologs Ssb1 and Ssb2 [21]. This would suggest that the putative function of NAC is masked by the redundancy of the CLIPS protein homeostasis network. Our analysis of the transcriptional response to NAC deletion (Figure 7F–G) provides insight into how the cellular circuitry compensates for the loss of NAC: A set of chaperones including both stress-inducible chaperones (e.g., Ssa2/4, Hsp42, and Hsp104) and CLIPS (e.g., Ssb1 and Ssb2), as well as several ribosomal proteins and assembly factors (Tables 3 and 4), were induced. This multifaceted response suggests that loss of NAC impairs protein folding and ribosome assembly. NAC has been proposed to have a role in mitochondrial targeting, as shown by a synthetic growth defect by deletion of cells lacking both Egd2 and the mitochondrial targeting factor Mft1 [19]. Our analysis revealed that mRNAs encoding mitochondrial proteins are enriched in association with both Btt1 and Egd2 (Figure 5A,B). Moreover we found that several proteins involved in mitochondrial assembly were induced in cells lacking NAC. Thus, a possible auxiliary role for NAC in cotranslational targeting polypeptides to the mitochondria deserves further investigation.

Is NAC a chaperone? Purified NAC does not prevent protein aggregation and NAC cannot bind directly to nascent chains unless they are ribosome associated [12],[39]. While this is unexpected for a traditional chaperone, NAC may be akin to trigger factor in bacteria, which interacts primarily with nascent chains in the context of the ribosome [76]. The distinct physicochemical properties of the nascent polypeptides associated with different NAC subunits may reflect the direct binding specificity of each individual subunit. A more detailed understanding of NAC substrate specificity must await better structural and biochemical understanding of this complex. The results of our global analysis will open the way for these experiments.

### Functional Interplay between NAC and SRP

The interplay between SRP and NAC has been controversial [12],[16],[17],[45],[77],[78]. In vitro experiments suggested that SRP can bind to cytosolic non-cognate nascent chains and that NAC and SRP can compete for RNC binding. On the other hand, in vivo analyses did not support the idea that NAC is required for proper SRP function and translocation [16]. Our experiments reconcile these observations and provide an integrated view of the regulation of SRP specificity by NAC. NAC modulates the interaction of SRP with nascent chains in vivo, favoring SRP binding to cognate substrates and disfavoring interactions with non-cognate targets (Figure 6F). Some ER-bound nascent proteins appear to depend on the presence of NAC in the cell for their interaction with SRP (NAC-dependent) while others do not (NAC-independent). Surprisingly, mRNA abundance and translation rate, rather than direct determinants of SRP affinity such as SS or TM hydrophobicity, are the major distinguishing features of the NAC-dependent versus the NAC-independent SRP interactions. This raises the idea that, in vivo, the specificity of the factors that interact with nascent proteins is governed not only by properties of the nascent polypeptide sequence, such as the intrinsic affinity of a given nascent chain for SRP, but also by the competition among cognate and non-cognate nascent polypeptides for these factors and by interactions between factors, exemplified by NAC and SRP.

Our analysis provides insight into the question of how signals such as SS or TM, which are recognized in a variable manner depending on affinity and concentration, can be read in the cell to determine a binary fate such as translocation, that is. proteins do or do not get translocated. Our data show that in wild type cells, SRP does bind with exquisite specificity to cognate substrates spanning a very wide range of cellular mRNA abundances, while disregarding very abundant cytosolic substrates that contain hydrophobic stretches with potential SRP-binding affinity. In ΔNAC cells, however, this specificity is relaxed, so that highly abundant non-cognate substrates bind to SRP and low abundance cognate substrates are lost from SRP. Thus NAC provides an additional level of specificity that fine-tunes SRP interactions to “sharpen” the response.

Our data can be explained in light of previous biochemical and biophysical measurements. NAC and SRP both contact the same ribosomal protein, L25, but have additional non-overlapping binding sites on the ribosome [11]. We observed that SRP-associated polysomes also contain associated NAC (Figure S6A). The interplay between these factors appears to be relevant for SRP specificity. SRP samples most translating ribosomes to encounter RNCs translating cognate polypeptides. Affinity measurements indicate that all translating ribosomes can bind SRP [58]. RNCs translating cytosolic polypeptides have significant affinity for SRP (ca. 8 nM) [58], however this interaction is salt sensitive and likely has a higher dissociation rate [79]. In contrast, SRP binds with extraordinarily high, subnanomolar affinity to RNCs bearing cognate substrates; this interaction is also salt-resistant, perhaps related to its low dissociation rate in vivo [80]. Of note, NAC was shown to reduce association of SRP to non-cognate RNCs. Accordingly, loss of NAC would result in a higher residence time for SRP on ribosomes translating highly abundant non-cognate mRNAs and a lower availability of SRP to bind low abundance cognate mRNAs. Our data suggesting that SRP and NAC overlap in binding to RNCs, much as proposed for trigger factor and SRP in bacteria, open the possibility for them acting in concert on a translating nascent chain. Because it appears that the conformational state of the ribosome contributes to SRP recruitment [43],[51], a more speculative possibility is that NAC exerts its regulatory activity through modulation of the ribosomal cycle.

### Robust Proofreading Mechanisms Ensure the Fidelity of ER Targeting

Despite the relaxed specificity of SRP binding to nascent chains in ΔNAC cells, there was no detectable difference in mRNA targeting to membranes in these cells, and no significant induction of a UPR response (Figure 7), supporting previous findings that NAC has no impact on translocation or the interaction of RNCs with membranes [77],[78]. This is likely the combined result of the redundancy of mRNA targeting pathways, which ensure that secretory proteins reach the membrane, together with proofreading mechanisms that prevent non-cognate SRP-RNC complexes from associating with membranes. For instance, the SRP targeting pathway contains an inbuilt proofreading mechanism at the SRP receptor (SR) level whereby the SRP-SR interaction is enhanced when SRP is bound to a signal sequence [81],[82]. Furthermore, the Sec61 translocon can stringently recognize signal sequence RNCs [5],[47]. These different mechanisms may together provide a robust system that ensures the fidelity of translocation even when the specificity of SRP interactions is impaired.

## Methods

### Strains, Protein, and RNA Affinity Purifications

Strains carrying chromosomally integrated Rpl16-TAP, Rpl17-TAP, Egd1-TAP, Egd2-TAP, Btt1-TAP, Srp68-TAP, and Srp72-TAP were obtained from Open Biosystems, Srp54-TAP from Euroscarf. *Δegd1* and *Δegd2* yeast strains from the *Saccharomyces* Genome Deletion Project [83] were used to obtain *Δegd1/Δegd2* by mating, sporulation, and tetrad dissection. *Sec65-1* strain was a kind gift of Peter Walter. Immunoaffinity purification of specific ribosome-associated factors together with ribosomes and associated RNAs was carried out exploiting the C-terminal TAP-tagged derivative of each selected protein [84]. Briefly, 1 liter cultures were grown to OD 0.7–0.8 in YPD. Following addition of cycloheximide (CHX) (0.1 mg/ml) to stabilize ribosome-nascent chain complexes, cells were harvested by centrifugation, washed twice in buffer A (50 mM Hepes-KOH [pH 7.5], 140 mM KCl, 10 mM MgCl2, 0.1% NP-40, 0.1 mg/ml CHX), resuspended in 2 ml of buffer B (buffer A plus 0.5 mM DTT, 1 mM PMSF, 20 µg/ml pepstatin A, 15 µg/ml leupeptin, 1 mM benzamidine, 10 µg/ml aprotinin, 0.2 mM AEBSF (Sigma), 0.2 mg/ml heparin, 50 U/ml Superasin (Ambion), and 50 U/ml RNAseOUT (Invitrogen)), and dripped into a conical 50 ml Falcon tube filled with and immersed in liquid nitrogen. Frozen cells were pulverized for 1 min at 30 Hz on a Retsch MM301 mixer mill. Pulverized cells were thawed and resuspended in 5 ml of buffer B; cell debris was removed by two sequential centrifugation at 8,000 g for 5 min at 4°C. A 100 µl aliquot (5%) of the supernatant was removed for reference RNA isolation. The remaining lysate was incubated with 6.7×10^6^ beads/µl of IgG-coupled magnetic beads (Dynabeads, Invitrogen) at 4°C for 2 h. Beads were washed once in 5 ml of buffer B for 2 min and 5 times in 1 ml buffer C (50 mM Hepes-KOH [pH 7.5], 140 mM KCl, 10 mM MgCl2, 0.01% NP-40, 10% glycerol, 0.5 mM DTT, 10 U/ml superasin, 10 U/ml RNAseOUT, 0.1 mg/ml CHX) for 1 min, resuspended in 100 µl of buffer C, and incubated for 2 h in 0.3 U/µl TEV protease (Invitrogen) at 16°C. Supernatant was recovered as final pulldown for protein and RNA isolation. Reference RNA was isolated using RNeasy mini kit (Quiagen), while RNA from the eluate was isolated by sequential extraction with Acid Phenol:Chloroform 125∶24∶1 (Ambion), Phenol/Chloroform/Isoamyl Alcohol 25∶24∶1 (Invitrogen), and chloroform followed by isopropanol precipitation with 15 µg of Glycoblue (Ambion) as carrier.

### Sucrose Density Fractionation

A total of 20 OD_254 nm_ were loaded on a 7%–47% sucrose gradient in buffer B without NP-40. The samples were centrifuged on a Beckman SW-41 rotor for 90 min at 42,000 rpm at 4°C. Gradients were continuously fractionated on an ISCO collector with a flow cell UV detector recording the absorbance at 254 nm. For protein detection by western blotting, fractions were precipitated with trichloroacetic acid, separated by SDS-PAGE, and analyzed by immunoblotting using the indicated antibodies. For RNA isolation and microarrays analysis, fractions corresponding to 60S, 80S, and polysomes were pooled to isolate polysome-associated RNA and the supernatant and low sucrose fractions were pooled to isolate free RNA. RNA was purified with RNeasy Mini Columns kit (Qiagen).

### Subcellular Fractionation and RNA Isolation

Free cytosolic polysomes and membrane-bound polysomes were fractionated by sedimentation velocity exactly as described [34],[85] starting with 250 ml of exponential growth cultures (of WT or *Δegd1/egd2*) in YPD. Total RNA from free and membrane-associated polysomes was purified with RNeasy Mini Columns Kit (Qiagen).

### Translocation Assays

50 ml cultures of WT, *Δegd1/egd2*, or *sec65-1* cells were grown in YPD at 30°C or followed by 1 h at 37°C (*sec65-1*). Cells were starved in SD-Met media for 30 min and labeled with ^35^S-methionine for 7 min. Endogenously expressed Kar2 and DPAP-B were immunoprecipitated with specific antibodies (a kind gift of Peter Walter and Mark Rose, respectively) and analyzed by SDS-PAGE in 8% acrylamide gels. Translocation defects were measured by comparing the ratio of non-processed precursor versus processed mature protein, namely non-signal sequence-cleaved versus cleaved Kar2 and an unglycosylated precursor versus glycosylated protein for DPAP-B.

### Microarray Sample Preparation

3 µg of reference RNA and 50% or up to 3 µg of TAP-tag affinity purified RNA were reverse transcribed with Superscript II (Invitrogen) in the presence of 5-(3-aminoallyl)-dUTP (Ambion) and dNTPs (Invitrogen) with a 1∶1 mixture of N9 and dT20V primers. The resulting cDNA was covalently linked to Cy3 (reference RNA) and Cy5 (purified RNA) NHS-monoesters (GE HealthSciences). Dye-labeled DNA was diluted into 20–40 µl solution containing 3× SSC, 25 mM Hepes-NaOH, pH 7.0, 20 µg poly(A) RNA (Sigma), and 0.3% SDS. The sample was incubated at 95°C for 2 min, spun at 14,000 rpm for 10 min in a microcentrifuge, and hybridized at 65°C for 12–16 h in the MAUI hybridization system (BioMicro). Following hybridization, microarrays were washed in 400 ml of four subsequent wash buffers made of 2×SSC with 0.05% SDS, 2×SSC, 1×SSC, and 0.2×SSC. The first wash was performed at 65°C for 5 min and the following washes for 2 min each at room temperature. Slides were briefly immersed in 95% ethanol and dried by centrifugation in a low-ozone environment to prevent Cy5/3 dyes destruction. Once dry, the microarrays were kept in a low-ozone environment during storage and scanning.

For fractionation experiments, 10 µg of free polysomes-RNA (Cy3) and 3 µg of rER polysomes-RNA were used for reverse transcription.

For analysis of transcriptional levels on mutant strains, 3 µg of reference RNA (wild type strain) (Cy3) and 3 µg of experimental RNA (mutant strain)(Cy5) were used for reverse transcription.

### Microarray Scanning, Processing, and Analysis

Microarrays were scanned with an Axon Instrument Scanner 4000B (Molecular Devices). PMP levels were adjusted to achieve 0.05% pixel saturation for IP experiments and 0% saturation for analysis of transcriptional levels. Data were collected with the GENEPIX 5.1 (Molecular Devices), and spots with abnormal morphology were excluded from further analysis. Arrays were computer normalized by the Stanford Microarray Database (SMD) [86]. Log_2_ median ratios were filtered for a regression correlation greater than 0.6 and a signal over background greater than 2.5 to remove low-confidence measurements. Hierachical clustering was performed with Cluster 3.0 [87], and results were visualized with Java TreeView [88].

At least three, usually four, independent biological replicates were employed for each condition. After removing features missing two or more values, we generated a representative dataset by running a one-class *t* test with 800 (SAM) [22]. Ultimately, substrates were defined as those encoded by mRNAs that were differentially expressed with a false discovery rate (FDR) (q-value) of 1.

### Data Analysis

Enriched GO terms among the identified targets were retrieved with GO Term Finder [89], which uses the hypergeometric density distribution function to calculate *p* values and the programs Genetrail [90] and FuncAssociate [91].

The GO database [89] was used to collect a list of GO categories. In these classifications, gene products can be affiliated with one or more GO category assignments.

Lists of proteins with predicted signal peptides and transmembrane regions were downloaded from the *Saccharomyces* Genome Database (SGD), which uses the prediction programs SignalIP [92] and TMHMM 2.0 [93], respectively.

Intrinsic disorder was predicted from the protein sequences with the Disopred2 software [94] after filtering out coiled-coil and transmembrane regions with the program pfilt (http://bioinf.cs.ucl.ac.uk/downloads/pfilt). Reported is the fraction of the protein sequence that is predicted to be unstructured. Sequence hydrophobicity was approximated by the average of the hydrophobicity profile, computed from the Kyte and Doolittle scale [95] with averaging over sliding windows of size 7. Hydrophobic stretches were defined as 5 or more consecutive amino acids that surpassed a threshold of 1 in the hydrophobicity profiles. Data on translation rate, ribosomal density, and mRNA expression were retrieved from [23] and [96]. Statistical data analysis was performed in R (www.r-project.org). Box plots indicate the data distribution through median, 25%, and 75% quartiles (filled box), as well as the range of non-outlier extremes (dashed lines).

## Supporting Information

Figure S1 Global strategy to define specificity of ribosome-associated factors. (A) TAP-tagged proteins associate with polysomes. The OD_254 nm_ profile (top) identifies the polysomal fractions on sucrose gradients. Individual fractions were analyzed for the presence of TAP-tagged proteins and the ribosomal protein Rpl3 by SDS-PAGE and immunoblotting. (B) Translational profile of yeast strains using sucrose gradients to recover polysome-bound mRNAs. Hierarchically clustered heat map of the translation profiles obtained from mRNA purified from polysomes. Three independent polysomes purifications were made from Rpl16-TAP tagged yeast strain. Pearson coefficient correlations between experiments are indicated on the tree. Significantly enriched GO terms (*p*<0.01) are indicated.
(TIF)

Figure S2 Using the SRP interactome to assess algorithms that identify transmembrane regions and signal sequences. (A) Prediction of SS or TM on the SRP enriched interactome. Number of SRP interactors with predicted transmembrane regions (i) or signal sequences (ii) using the indicated prediction programs. The line represents the consensus, that is, the proteins predicted to have a transmembrane region in all three programs. (B) Prediction on the unenriched non-SRP interactors. Number of proteins unenriched in SRP pulldowns (non-SRP interactors) with predicted transmembrane regions (i) or signal sequences (ii). The line represents the consensus, that is, the proteins predicted to have a transmembrane region in all three programs.
(TIF)

Figure S3 SRP enrichment shows weak correlation with hydrophobicity. (A) Correlation between SRP enrichment (as SAM score) and N-terminus hydrophobicity of the corresponding protein. Genes were grouped by SAM score in bins of 1. Hydrophobicity was calculated as an average of the score obtained with the Kyte-Doolittle algorithm for the first 50 aminoacids of the protein corresponding to every grouped gene, with a window of 11. (B) Scatterplot showing the correlation between SRP binding (as SAM score) and N-terminus hydrophobicity, calculated as in (A). (C) Scatterplot showing the correlation between SRP binding (as SAM score) and maximum hydrophobicity of the signal sequence (SS) or transmembrane region (TM) on proteins with these features.
(TIF)

Figure S4 Scatterplots comparing the enrichment obtained for membrane and SRP-associated mRNAs encoding proteins of different subcellular localizations.
(TIF)

Figure S5 Intact polysomes required for Egd2-TAP association with mRNAs. (A) Cycloheximide omission causes polysome dissociation and Egd2 release. Polysome profiles of cell extracts were prepared in the presence (+ CHX) or absence (−CHX) of cycloheximide and fractionated by sucrose gradient centrifugation. The OD254 nm profile (top) identifies the ribosome elution profiles across the sucrose gradient. In the absence of CHX, a large fraction of polysomes dissociate yielding monosomes. Individual fractions were analyzed for the presence of Egd2 and the ribosomal protein Rpl3 by SDS-PAGE and immunoblotting. The migration of Egd2/αNAC into the heavier fractions of the gradient depends on its association with translating polysomes. (B) Amount of total RNA recovered by immunopurification of Egd2-TAP from +CHX and −CHX cells. The same amount of cells was used for both conditions. Omitting CHX leads to a substantial reduction in the total amount of total RNA recovered, consistent with a substantial reduction in Egd2 binding to ribosomes. (C) Number of significant mRNAs that are significantly associated with Egd2 in the presence (+CHX) or absence (−CHX) of cycloheximide. Omission of CHX dramatically reduces the number of mRNAs significantly (1% FDR) associated with Egd2. Most mRNAs associated with Edg2 in −CHX also bind +CHX, suggesting they arise from residual RNCs that did not dissociate.
(TIF)

Figure S6 Effect of NAC deletion on the SRP interactome. (A) NAC and SRP associate to the same ribosomal complexes. Eluates obtained after immunopurification of SRP using the indicated TAP-tagged SRP subunits, Srp54p, Srp68p, and Srp72p, were analyzed by immunoblot for the presence of an NAC with anti-Egd2 polyclonal antibody and for the presence of ribosomes using anti-Rpl3 monoclonal antibody. (B) SRP associates with polysomes in WT and ΔNAC strains. Yeast extracts from the indicated cells were fractionated on sucrose gradients and individual fractions were analyzed for the presence of SRP and the ribosomal protein Rpl3. The OD_254 nm_ profile (top) identifies the polysomal fractions.
(TIF)

Figure S7 Effects of NAC deletion (*Δegd2/Δegd1*) on the SRP interactome. (A) Scatterplot showing enrichment of SRP-associated mRNAs in WT and ΔNAC strains. Purple, NAC-independent SRP interactors; blue, NAC-dependent SRP interactors; green, Off target SRP interactors. Values obtained for two known SRP targets, Kar2 and DPAP-B, are shown. (B) Comparison of the fraction of SRP-associated mRNAs encoding proteins of different subcellular localizations in the WT (black), ΔNAC (white), or genome (grey). (C) Distribution of lengths of first hydrophobic stretches found in mRNAs encoding targets of different SRP interactors and the genome. To look for hydrophobic stretches, protein sequences were analyzed to look for groups of 5 or more aminoacids with a Kyte-Doolittle scale value higher than 1 using a sliding window of 7.
(TIF)

Figure S8 Effects of NAC' deletion (*Δegd2/Δbtt1*) on the SRP interactome. (A) Hierarchically clustered heat map of the SRP interactomes in either WT (four independent replicates) or NAC'-deleted (ΔNAC') (three independent replicates) cells. Boxes indicate genes significantly enriched (*p*<0.01) in the SRP-interactome in both strains (purple) (NAC-independent) or only in ΔNAC' cells (green) (Off-target) or WT cells (blue) (NAC-dependent). GO ontology categories significantly enriched (*p*<0.01) in each dataset are indicated. Pearson coefficient correlations are indicated on the tree. (B) Comparison of fraction of SRP substrates with predicted signal sequences (from Signal IP) or transmembrane regions (from TMHMM) in WT cells (black), ΔNAC cells (*Δegd2/Δegd1*) (white), ΔNAC' (*Δegd2/Δbtt1*) (dark grey), or the yeast genome (grey). (C) Comparison of the fraction of SRP-associated mRNAs encoding proteins of different subcellular localizations in the WT (black), ΔNAC (white), ΔNAC' (dark grey), or the yeast genome (grey). (D) The differences observed between the SRP interactors in WT, ΔNAC, and ΔNAC' are independent of the statistical threshold employed. Fraction of SRP-associated nascent chains encoding proteins with predicted Signal Sequences (SS) or Transmembrane Regions (TM) in the WT, ΔNAC, and ΔNAC' backgrounds, calculated as in (B) but using different statistical stringencies. The graph shows the percentage of significant SRP targets with TM or SS in datasets obtained using the indicated statistical thresholds for FDR (False Discovery Rates). WT (black), ΔNAC (white), and ΔNAC' (dark grey).
(TIF)

Table S1 SRP interacting polypeptides without SS/TM domains that are encoded by membrane-associated mRNAs.
(DOC)

## Abbreviations

CLIPS: Chaperones Linked to Protein Synthesis
FDR: false discovery rate
IP: immunopurification
NAC: nascent chain associated complex
RBPs: RNA-binding proteins
RNC: ribosome-nascent chain complex
SR: SRP receptor
SRP: Signal Recognition Particle
SS: signal sequence
TAP: Tandem-Affinity-Purification
TM: transmembrane domain
UPR: unfolded protein response
