# Supplementary figures and images for: Defining the Specificity of Cotranslationally Acting Chaperones by Systematic Analysis of mRNAs Associated with Ribosome-Nascent Chain Complexes
Source: PLoS Biol. 2011 Jul 12;9(7):e1001100. doi: 10.1371/journal.pbio.1001100 (PMC3134442; doi:10.1371/journal.pbio.1001100)

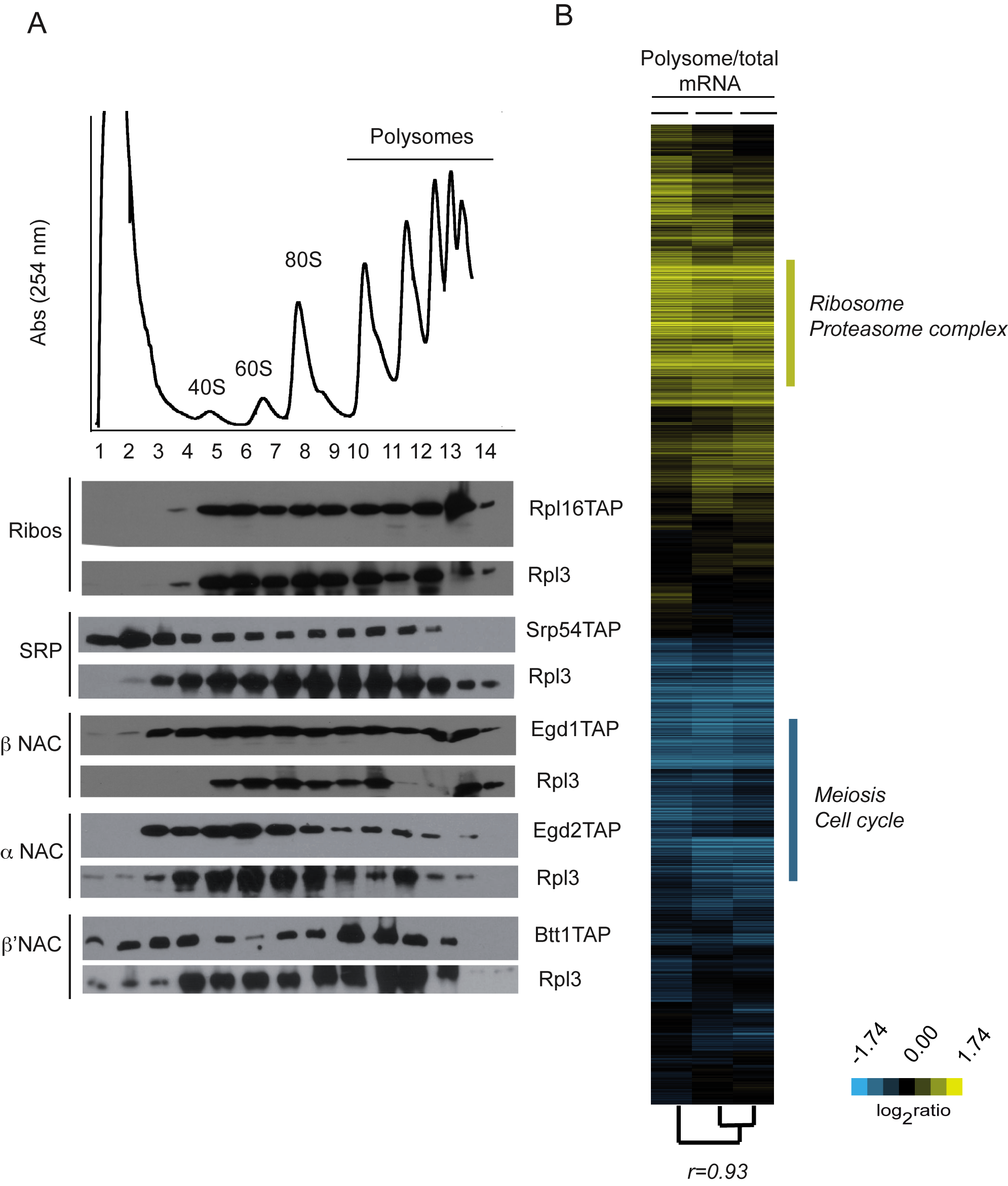

Supplement: Figure S1 — Global strategy to define specificity of ribosome-associated factors. (A) TAP-tagged proteins associate with polysomes. The OD254 nm profile (top) identifies the polysomal fractions on sucrose gradients. Individual fractions were analyzed for the presence of TAP-tagged proteins and the ribosomal protein Rpl3 by SDS-PAGE and immunoblotting. (B) Translational profile of yeast strains using sucrose gradients to recover polysome-bound mRNAs. Hierarchically clustered heat map of the translation profiles obtained from mRNA purified from polysomes. Three independent polysomes purifications were made from Rpl16-TAP tagged yeast strain. Pearson coefficient correlations between experiments are indicated on the tree. Significantly enriched GO terms (p<0.01) are indicated. (TIF) [file pbio.1001100.s001.tif]

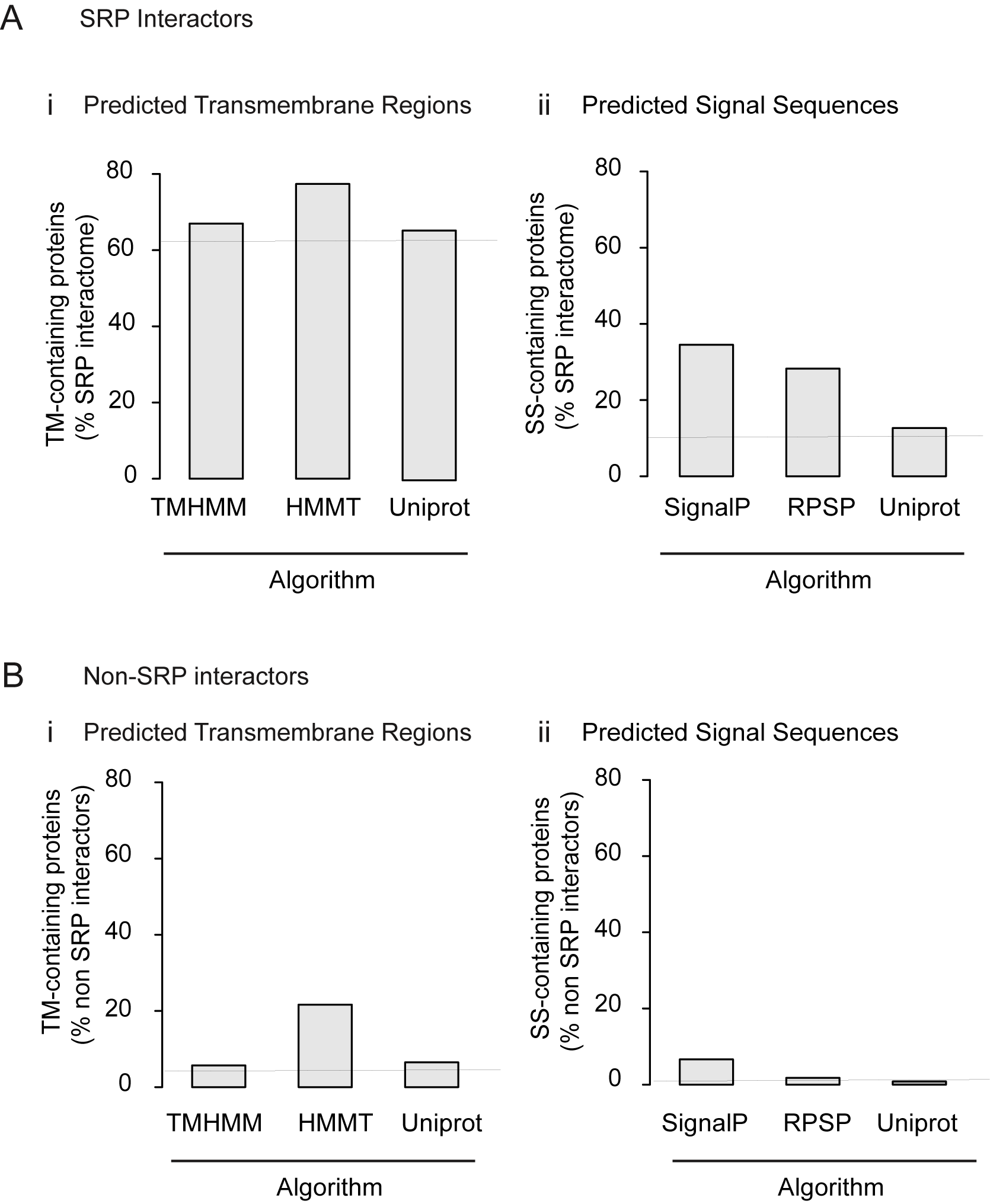

Supplement: Figure S2 — Using the SRP interactome to assess algorithms that identify transmembrane regions and signal sequences. (A) Prediction of SS or TM on the SRP enriched interactome. Number of SRP interactors with predicted transmembrane regions (i) or signal sequences (ii) using the indicated prediction programs. The line represents the consensus, that is, the proteins predicted to have a transmembrane region in all three programs. (B) Prediction on the unenriched non-SRP interactors. Number of proteins unenriched in SRP pulldowns (non-SRP interactors) with predicted transmembrane regions (i) or signal sequences (ii). The line represents the consensus, that is, the proteins predicted to have a transmembrane region in all three programs. (TIF) [file pbio.1001100.s002.tif]

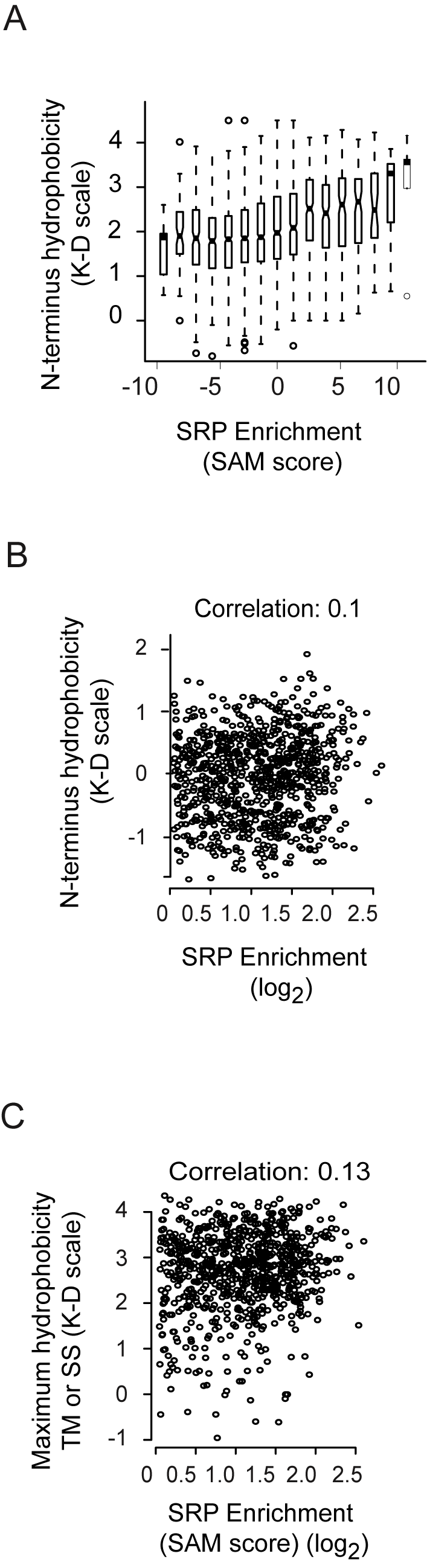

Supplement: Figure S3 — SRP enrichment shows weak correlation with hydrophobicity. (A) Correlation between SRP enrichment (as SAM score) and N-terminus hydrophobicity of the corresponding protein. Genes were grouped by SAM score in bins of 1. Hydrophobicity was calculated as an average of the score obtained with the Kyte-Doolittle algorithm for the first 50 aminoacids of the protein corresponding to every grouped gene, with a window of 11. (B) Scatterplot showing the correlation between SRP binding (as SAM score) and N-terminus hydrophobicity, calculated as in (A). (C) Scatterplot showing the correlation between SRP binding (as SAM score) and maximum hydrophobicity of the signal sequence (SS) or transmembrane region (TM) on proteins with these features. (TIF) [file pbio.1001100.s003.tif]

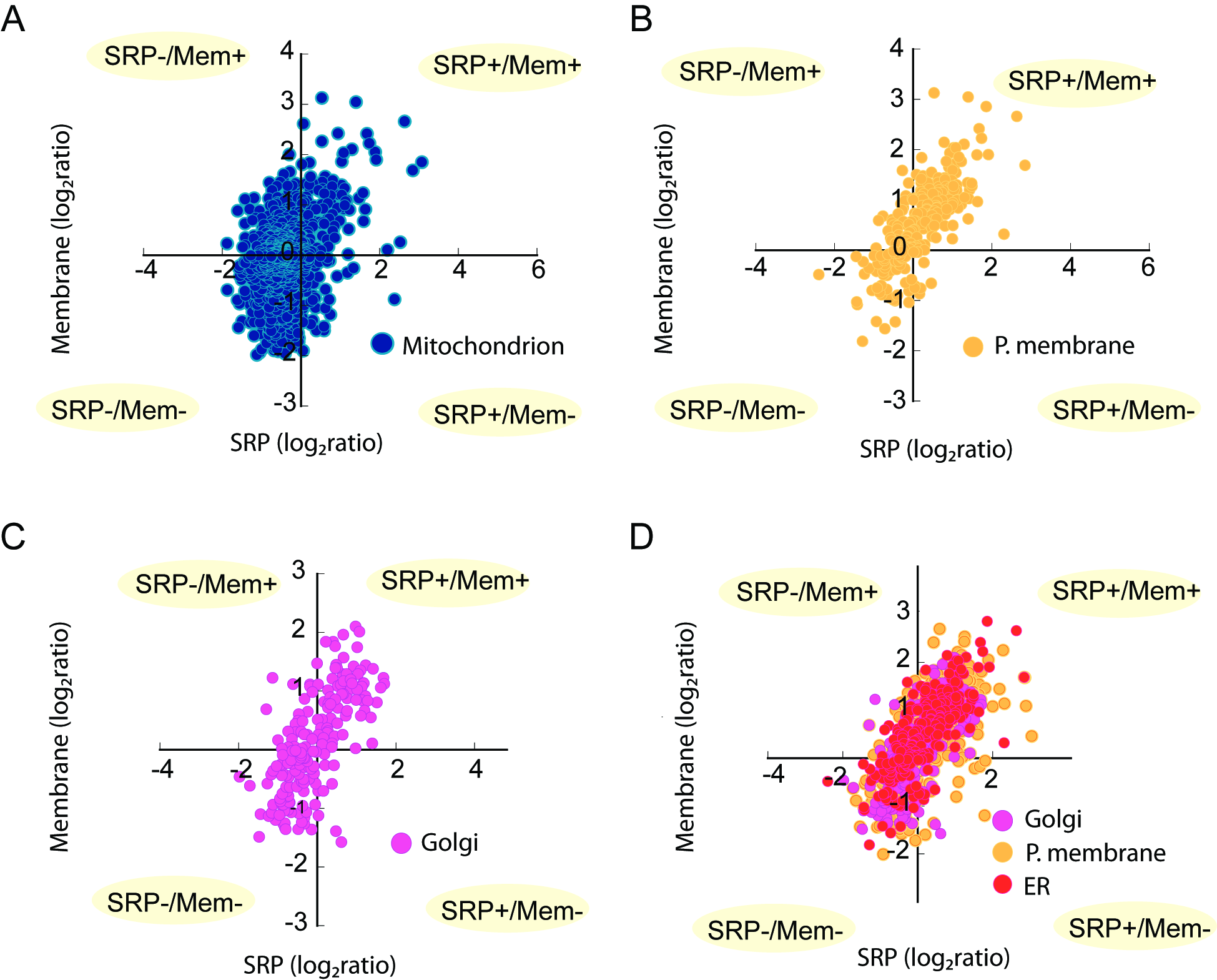

Supplement: Figure S4 — Scatterplots comparing the enrichment obtained for membrane and SRP-associated mRNAs encoding proteins of different subcellular localizations. (TIF) [file pbio.1001100.s004.tif]

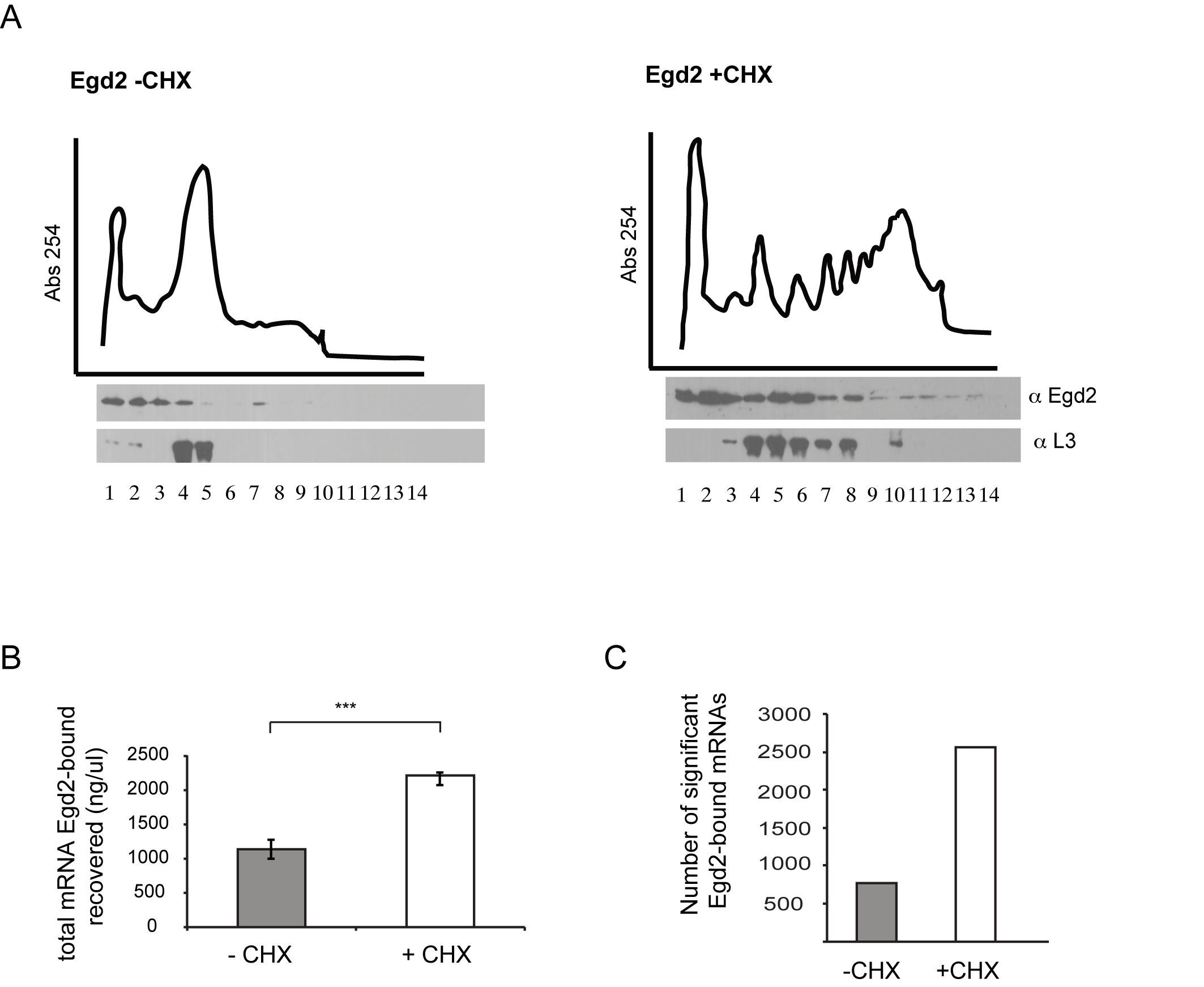

Supplement: Figure S5 — Intact polysomes required for Egd2-TAP association with mRNAs. (A) Cycloheximide omission causes polysome dissociation and Egd2 release. Polysome profiles of cell extracts were prepared in the presence (+ CHX) or absence (−CHX) of cycloheximide and fractionated by sucrose gradient centrifugation. The OD254 nm profile (top) identifies the ribosome elution profiles across the sucrose gradient. In the absence of CHX, a large fraction of polysomes dissociate yielding monosomes. Individual fractions were analyzed for the presence of Egd2 and the ribosomal protein Rpl3 by SDS-PAGE and immunoblotting. The migration of Egd2/αNAC into the heavier fractions of the gradient depends on its association with translating polysomes. (B) Amount of total RNA recovered by immunopurification of Egd2-TAP from +CHX and −CHX cells. The same amount of cells was used for both conditions. Omitting CHX leads to a substantial reduction in the total amount of total RNA recovered, consistent with a substantial reduction in Egd2 binding to ribosomes. (C) Number of significant mRNAs that are significantly associated with Egd2 in the presence (+CHX) or absence (−CHX) of cycloheximide. Omission of CHX dramatically reduces the number of mRNAs significantly (1% FDR) associated with Egd2. Most mRNAs associated with Edg2 in −CHX also bind +CHX, suggesting they arise from residual RNCs that did not dissociate. (TIF) [file pbio.1001100.s005.tif]

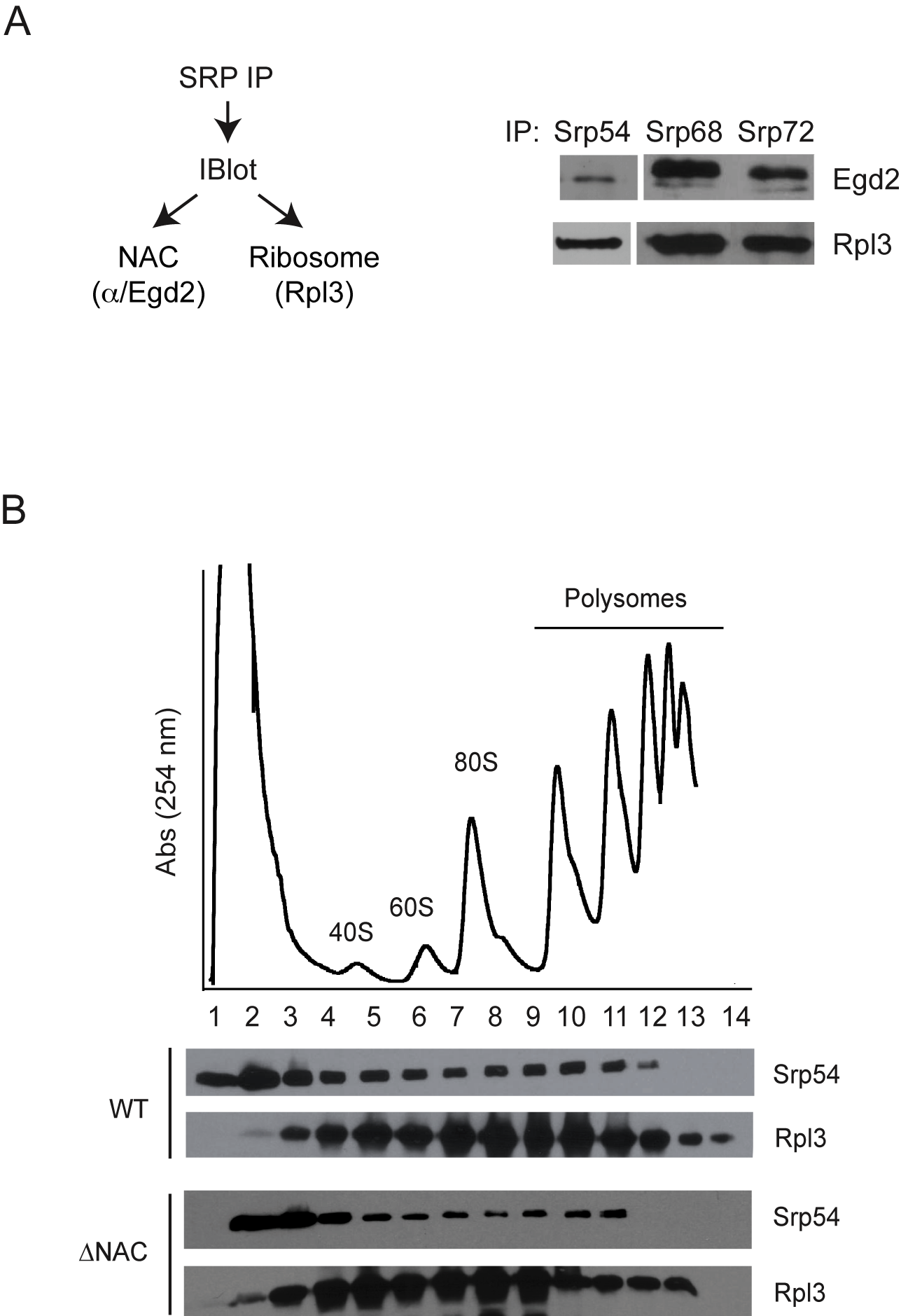

Supplement: Figure S6 — Effect of NAC deletion on the SRP interactome. (A) NAC and SRP associate to the same ribosomal complexes. Eluates obtained after immunopurification of SRP using the indicated TAP-tagged SRP subunits, Srp54p, Srp68p, and Srp72p, were analyzed by immunoblot for the presence of an NAC with anti-Egd2 polyclonal antibody and for the presence of ribosomes using anti-Rpl3 monoclonal antibody. (B) SRP associates with polysomes in WT and ΔNAC strains. Yeast extracts from the indicated cells were fractionated on sucrose gradients and individual fractions were analyzed for the presence of SRP and the ribosomal protein Rpl3. The OD254 nm profile (top) identifies the polysomal fractions. (TIF) [file pbio.1001100.s006.tif]

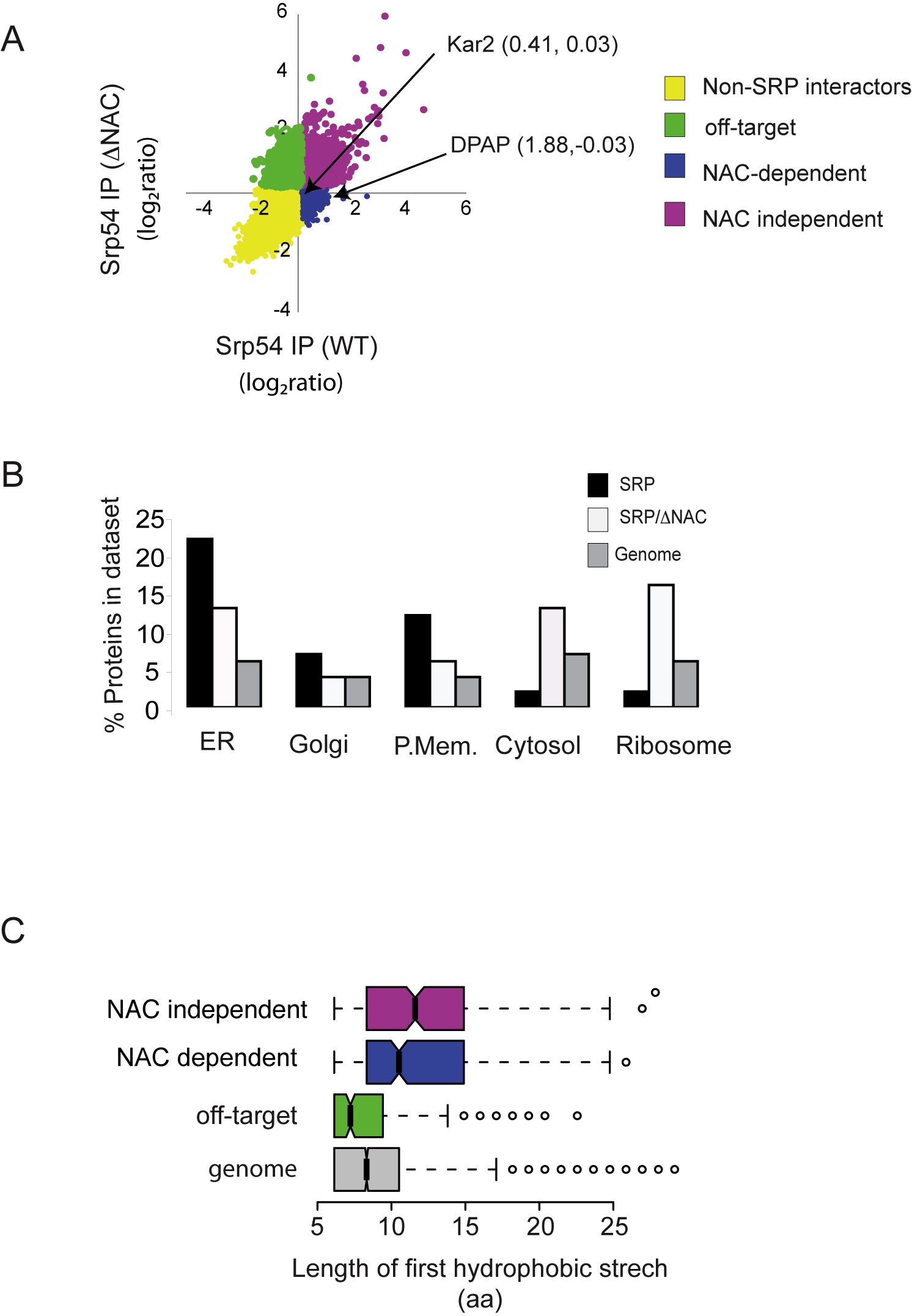

Supplement: Figure S7 — Effects of NAC deletion (Δegd2/Δegd1) on the SRP interactome. (A) Scatterplot showing enrichment of SRP-associated mRNAs in WT and ΔNAC strains. Purple, NAC-independent SRP interactors; blue, NAC-dependent SRP interactors; green, Off target SRP interactors. Values obtained for two known SRP targets, Kar2 and DPAP-B, are shown. (B) Comparison of the fraction of SRP-associated mRNAs encoding proteins of different subcellular localizations in the WT (black), ΔNAC (white), or genome (grey). (C) Distribution of lengths of first hydrophobic stretches found in mRNAs encoding targets of different SRP interactors and the genome. To look for hydrophobic stretches, protein sequences were analyzed to look for groups of 5 or more aminoacids with a Kyte-Doolittle scale value higher than 1 using a sliding window of 7. (TIF) [file pbio.1001100.s007.tif]

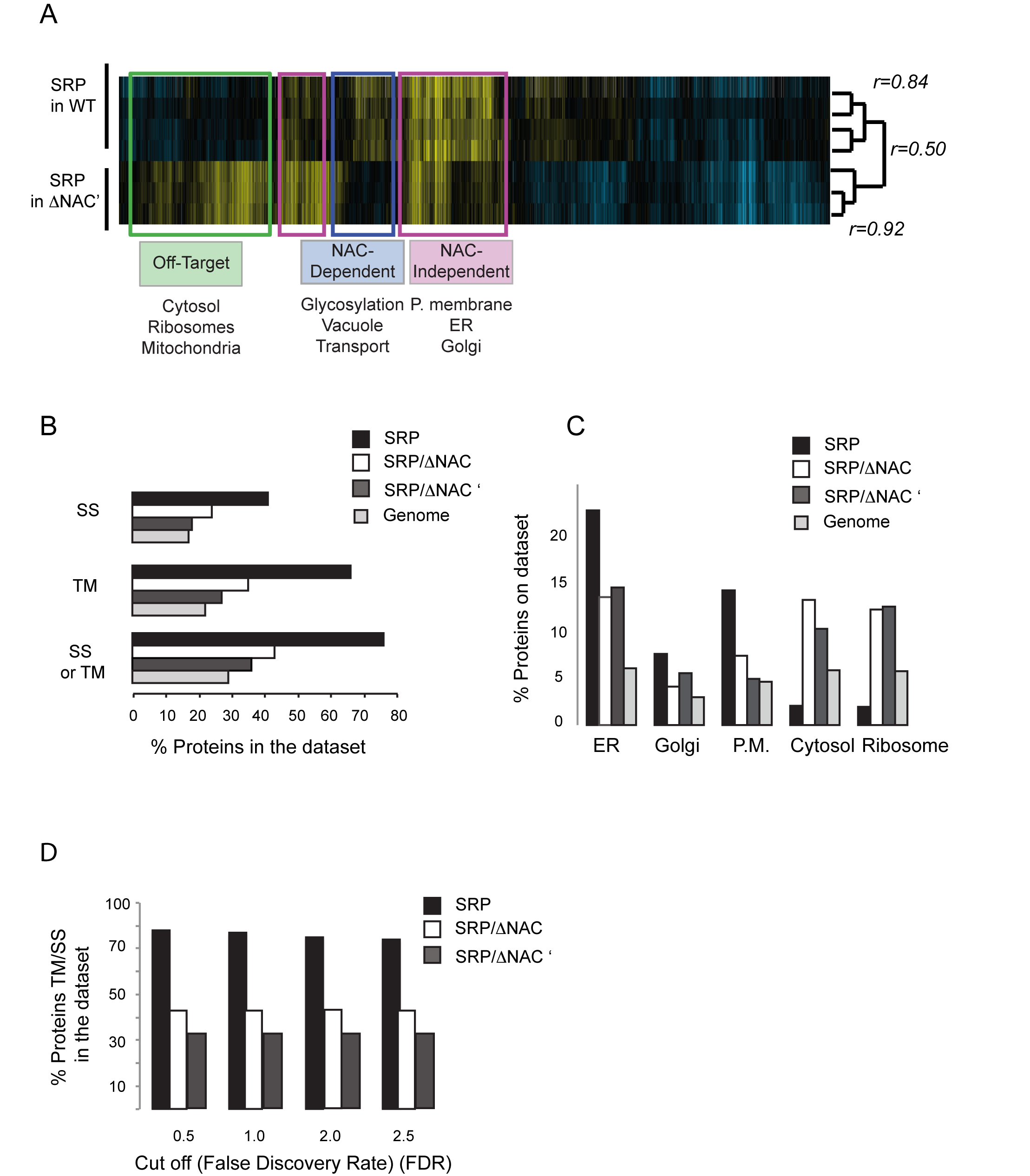

Supplement: Figure S8 — Effects of NAC' deletion (Δegd2/Δbtt1) on the SRP interactome. (A) Hierarchically clustered heat map of the SRP interactomes in either WT (four independent replicates) or NAC'-deleted (ΔNAC') (three independent replicates) cells. Boxes indicate genes significantly enriched (p<0.01) in the SRP-interactome in both strains (purple) (NAC-independent) or only in ΔNAC' cells (green) (Off-target) or WT cells (blue) (NAC-dependent). GO ontology categories significantly enriched (p<0.01) in each dataset are indicated. Pearson coefficient correlations are indicated on the tree. (B) Comparison of fraction of SRP substrates with predicted signal sequences (from Signal IP) or transmembrane regions (from TMHMM) in WT cells (black), ΔNAC cells (Δegd2/Δegd1) (white), ΔNAC' (Δegd2/Δbtt1) (dark grey), or the yeast genome (grey). (C) Comparison of the fraction of SRP-associated mRNAs encoding proteins of different subcellular localizations in the WT (black), ΔNAC (white), ΔNAC' (dark grey), or the yeast genome (grey). (D) The differences observed between the SRP interactors in WT, ΔNAC, and ΔNAC' are independent of the statistical threshold employed. Fraction of SRP-associated nascent chains encoding proteins with predicted Signal Sequences (SS) or Transmembrane Regions (TM) in the WT, ΔNAC, and ΔNAC' backgrounds, calculated as in (B) but using different statistical stringencies. The graph shows the percentage of significant SRP targets with TM or SS in datasets obtained using the indicated statistical thresholds for FDR (False Discovery Rates). WT (black), ΔNAC (white), and ΔNAC' (dark grey). (TIF) [file pbio.1001100.s008.tif]
