# Supplementary material for: Defining the Specificity of Cotranslationally Acting Chaperones by Systematic Analysis of mRNAs Associated with Ribosome-Nascent Chain Complexes
Source: PLoS Biol. 2011 Jul 12;9(7):e1001100. doi: 10.1371/journal.pbio.1001100 (PMC3134442; doi:10.1371/journal.pbio.1001100)
Supplement: Table S1 — SRP interacting polypeptides without SS/TM domains that are encoded by membrane-associated mRNAs. (DOC) [file pbio.1001100.s009.doc]

**Table S1. SRP interacting polypeptides without SS/TM domains that are encoded by membrane-associated mRNAs.**

| **GO term** | **Frequency** | **Genes** |
| --- | --- | --- |
| Mitochondrion | (7) 13.5 % | CIT2,ENO1,ENO2,OPI3,YKL070W,IRA2,GIP3 |
| Nucleus | (9) 17.3% | YBL005WA,CDC27,SCC2,GCN4, HAC1,YGR109WA, YJR027W,RGT1,SGO1 |
| Endoplasmic reticulum | (6) 11.5% | SED4,LCB2,SRP101,DPL1,ERG5,GIP3 |
| Cytosol | (2) 3.8% | ENO1,ENO2 |
| Unknown | (21) 40.4% | YBR206W,YCR025C,BSC1,YEL076C,ROG3, YGL199C,YGR018C,YHL049C,YIL082W, YIR044C,YJL211C,YJR107W,YJR146W, YLR463C,BSC3,YML002W,YMR013WA, YNL276C, YOR093C,YOR248W,YPR195C |

Membrane-associated mRNAs and messengers enriched in SRP pulldowns lacking SS or TM were compared to generate a list of overlapping messengers. 52 total SRP targets without SS/TM are enriched in the membrane fraction. Assignment of the corresponding location was made retrieving GO ontology (component) categories from SGD.
